# Long Slender Piezo-Resistive Silicon Microprobes for Fast Measurements of Roughness and Mechanical Properties inside Micro-Holes with Diameters below 100 µm

**DOI:** 10.3390/s19061410

**Published:** 2019-03-22

**Authors:** Uwe Brand, Min Xu, Lutz Doering, Jannick Langfahl-Klabes, Heinrich Behle, Sebastian Bütefisch, Thomas Ahbe, Erwin Peiner, Stefan Völlmeke, Thomas Frank, Bodo Mickan, Ilia Kiselev, Michael Hauptmannl, Michael Drexel

**Affiliations:** 1 Physikalisch-Technische Bundesanstalt (PTB), Bundesallee 100, D-38116 Braunschweig, Germany; Min.Xu@ptb.de (M.X.); Lutz.Doering@ptb.de (L.D.); Jannick.Langfahl-Klabes@ptb.de (J.L.-K.); Heinrich.Behle@ptb.de (H.B.); Sebastian.Buetefisch@ptb.de (S.B.); Thomas.Ahbe@ptb.de (T.A.); Bodo.Mickan@ptb.de (B.M.); 2 Institute of Semiconductor Technology (IHT), Technische Universität Braunschweig, Hans-Sommer-Straße 66, D-38106 Braunschweig, Germany; e.peiner@tu-braunschweig.de; 3 Laboratory for Emerging Nanometrology (LENA), Technische Universität Braunschweig, Langer Kamp 6, D-38106 Braunschweig, Germany; 4 CiS Forschungsinstitut für Mikrosensorik GmbH, Konrad-Zuse-Straße 14, D-99099 Erfurt, Germany; svoellmeke@cismst.de (S.V.); tfrank@cismst.de (T.F.); 5 Breitmeier Messtechnik GmbH, Englerstr. 24, D-76275 Ettlingen, Germany; Kiselev@breitmeier.de (I.K.); Hauptmannl@breitmeier.de (M.H.); drexel@breitmeier.de (M.D.)

**Keywords:** cantilever microprobe, high-speed, contact resonance, tip wear, piezo-resistive, mechanical damping, tip-testing standard

## Abstract

During the past decade, piezo-resistive cantilever type silicon microprobes for high-speed roughness measurements inside high-aspect-ratio microstructures, like injection nozzles or critical gas nozzles have been developed. This article summarizes their metrological properties for fast roughness and shape measurements including noise, damping, tip form, tip wear, and probing forces and presents the first results on the measurement of mechanical surface parameters. Due to the small mass of the cantilever microprobes, roughness measurements at very high traverse speeds up to 15 mm/s are possible. At these high scanning speeds, considerable wear of the integrated silicon tips was observed in the past. In this paper, a new tip-testing artefact with rectangular grooves of different width was used to measure this wear and to measure the tip shape, which is needed for morphological filtering of the measured profiles and, thus, for accurate form measurements. To reduce tip wear, the integrated silicon tips were replaced by low-wear spherical diamond tips of a 2 µm radius. Currently, a compact microprobe device with an integrated feed-unit is being developed for high-speed roughness measurements on manufacturing machines. First measurements on sinusoidal artefacts were carried out successfully. Moreover, the first measurements of the elastic modulus of a polymer surface applying the contact resonance measurement principle are presented, which indicates the high potential of these microprobes for simultaneous high-speed roughness and mechanical parameter measurements.

## 1. Introduction

The dimensional quality control of microstructures is mostly undertaken by optical microscopy, micro-coordinate measuring machines, or if nm lateral and vertical resolution is needed, by scanning probe microscopy, which is also known as atomic force microscopy (AFM). For more difficult tasks like measurements at vertical sidewalls, special AFM cantilevers have been developed [1]. For the further development of fuel injection nozzles, the challenge of measuring the shape of ever smaller injection holes and the roughness inside the holes has arisen. To measure the form of these micro-nozzles, several micro-coordinate measuring machines (µCMM) with probing sphere radii down to 10 µm were developed [2]. To also traceably measure the fine roughness below 0.3 µm [3] inside these narrow nozzles with diameters below 100 µm, probes with probing tip radii of 2 µm are needed [4]. Since these could not be realized by µCMMs, long slender piezo-resistive silicon cantilevers with integrated silicon tips with radii between 0.05 µm and 1 µm were developed for that purpose [5]. Surface roughness and the shape and form of small injection nozzles could be measured down to diameters of 100 µm [6] with these microprobes. Besides the capability to measure inside micro-nozzles, these light weight sensors have the potential to deliver very fast measurements, since their mass (0.12 mg) is orders of magnitude smaller than that of conventional roughness probes (1 g). The first fast measurements with traverse speeds up to 15 mm/s were successfully made on roughness standards [7].

Due to the prospect of high scanning speeds, these sensors have aroused the interest of industry, which needs high-speed tactile measurements to improve quality control directly on machines such as micro-finishing machines, tribological measurement systems, or large roll measuring machines [8]. Due to high vibration levels on these machines, the mechanical damping of the microprobes has to be improved.

For measurements at high traverse speeds, it is important to measure at high sampling rates. This implies the usage of higher signal bandwidths and, hence, the noise behavior of the sensor has to be investigated. Moreover, sensor noise in the dependence of scanning speed and probing force needs to be determined. Another challenge for industrial shape measurements is the desired uncertainty of 50 nm. In order to achieve this goal, it is necessary to remove the influence of the tip radius on the measured profiles by morphological filtering. Thus, reliable methods to measure the tip shape of microprobes are required. A recently developed tip-testing artefact [9] seems to be well suited to determine the tip shape and opening angle of the tips.

At fast roughness measurements, a continuous small wear of the integrated silicon tip was observed [7]. To overcome the wear problem, two methods are tested here: a hard coating of the tips and the gluing of full diamond tips to the probes. 

For fast tactile measurements, the exact knowledge of the probing force is very important for non-destructive measurements. For this purpose, simple and reliable methods based on standards or sensors are required.

Section 2 describes experimental details in addressing the scientific questions outlined above. In Section 3, details of experimental equipment are presented and, in Section 4, two different applications of the microprobes are discussed.

## 2. Metrological Properties of Piezo-Resistive Silicon Microprobes

Long piezo-resistive cantilever micro-probes for the measurement inside of small holes were first developed by Braunschweig’s Technical University [10,11]. The Physikalisch-Technische Bundesanstalt (PTB) first used these 5 mm long sensors, which initially did not contain an integrated stylus tip, to measure the probing force of stylus instruments [12]. As part of a joint project entitled μgeoMess, these micro-probes have been further developed with the aim of measuring the roughness and shape inside diesel injection nozzles [13]. The sensors developed have since been available from the CiS Research Institute for Microsensors in Erfurt [14].

The sensor components are the long rectangular cantilever with an integrated silicon tip at the end with the four doped piezo-resistors connected to a Wheatstone bridge at the front end of the beam for deflection sensing and large electrical contacts.

**Figure 1:**
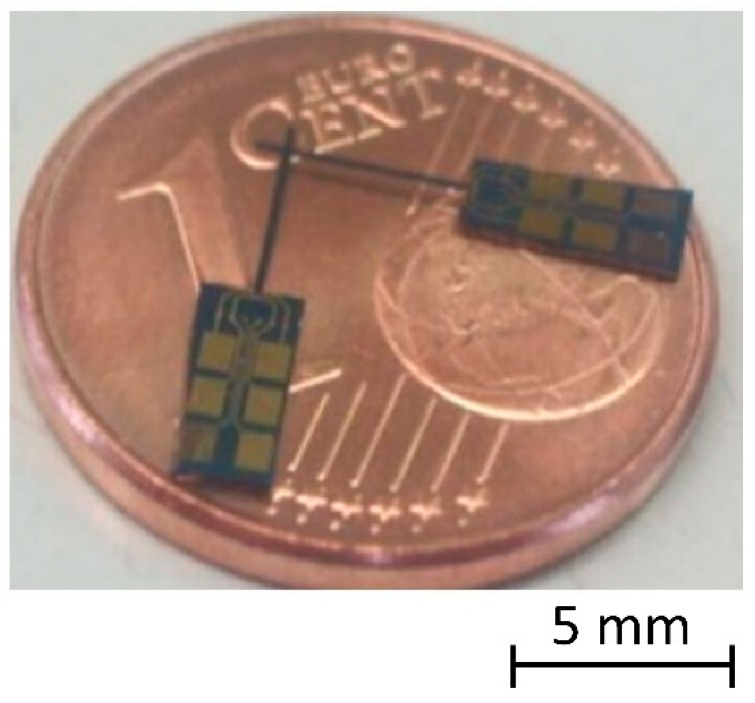
Two 5-mm long piezo-resistive silicon microprobes with integrated silicon tip on a 1 Euro Cent coin.

Three different geometries of sensors were designed and were made available by CiS. The largest micro-probe has dimensions (length × width × thickness) of 5 mm × 200 μm × 50 μm (see Figure 1), the second type has dimensions of 3 mm × 100 µm × 25 µm, and the smallest micro-probe has dimensions of 1.5 mm × 30 μm × 25 μm [15]. The smallest one was designed for very narrow injections nozzles with bore-hole diameters of less than 100 µm diameter. Since it is only 30 µm wide, it was necessary to increase the width of the beam at the very beginning, in order to have enough space for the four piezo-resistors. Thus, the effective length of the smallest microprobe is only 1.25 mm. The two other types were designed for measurements inside holes with larger diameters and larger lengths such as critical flow Venturi nozzles. So far, only the 5-mm and 1.25-mm in length microprobes have been investigated since the 3-mm microprobes do not offer further advantages compared to the other two.

The micro probes are manufactured by volume micro processes on 4-inch wafers out of (100) n-doped silicon. There are four major manufacturing steps: the creation of the piezo-resistive Wheatstone bridge, the etching of the micro-tip, the metallization of the electrical contacts, and the release of the micro-cantilever. To realize integrated micro-tips with heights between 10 µm and 200 μm with almost conical shape, wet-chemically etching with a potassium hydroxide solution (KOH) was used. For the measurement of roughness, according to ISO 3274 [4], a robust tip with a radius of curvature of 2 μm and an opening angle of 60°, or respectively, of 90° is recommended. 

Other outstanding features of the micro-probes are their high strain gauge factors of K ≈ 80 and the very low power loss.

### 2.1. Noise

The noise of a profile measuring system depends on many influencing factors. The most important noise sources are sensor noise, scanning table noise, and environmental noise such as vibrations, electromagnetic, and acoustic noise. Therefore, the static noise of a 5-mm in length microprobe, without any contact with a surface, i.e., a free-hanging microprobe was measured first. PTB uses a traceable system called the Profilescanner for topography measurements with the microprobe (see Section 3.1). Thus, noise measurements are shown for free-hanging microprobes on the Profilescanner. For the measurement of very long gas nozzles, longer microprobes were fabricated simply by gluing a second 5-mm-long cantilever onto the back of a microprobe. For comparison, for these enlarged microprobes, the static noise will be presented. The noise behavior of the 1.25-mm long microprobe is not shown here since it is comparable to that of the 5-mm long microprobe.

Since different surface topographies exert different accelerations on the probing tip during scanning measurements, these produce different vibrations of the microprobe cantilever. These vibrations will be quickly damped out, depending on the damping coefficient of the microprobe (see Section 2.2). Furthermore, the mechanical contact of the microprobe to a surface leads to a damping of microprobe oscillations. As an example, the free oscillation amplitude of the microprobe of 2 µm is attenuated by the elastic contact with a surface down to 200 nm.

To investigate the effect of an increasing scanning speed on measurement noise, two specimens were investigated for different scanning speeds. Roughness measurements at different scanning speeds on an optical flat and on a roughness standard were investigated. Both were measured as they are and with an inclination of 100 µm to simulate the influence of increasing probing force since the excited oscillation amplitudes should be proportional to the static probing force.

#### 2.1.1. Static Microprobe Noise

The sensor noise of a 5-mm in length piezo-resistive silicon microprobe was measured by Peiner et al. [13]. A noise floor of 10 nV/$\sqrt{\mathrm{Hz}}$ above 100 Hz was obtained. Thus, at a sensitivity of 200 nV/nm, a noise level of 50 pm/$\sqrt{\mathrm{Hz}}$ was obtained.

#### 2.1.2. Static Noise of Microprobe on Measuring Machine Profilescanner

**Figure 2:**
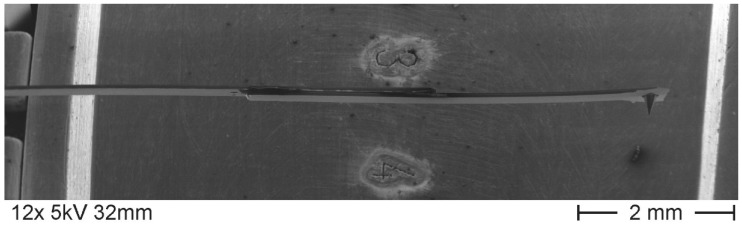
Extended microprobe of 7.5-mm length consisting of two 5-mm in length cantilevers glued to each other.

The noise of the piezo-resistive silicon microprobe on the Profilescanner, PTB’s measuring system, was measured under different environmental disturbing influences for two different sensors. The noise of a conventional 5-mm in length microprobe was compared with that of a 7.5-mm in length microprobe. The 7.5-mm long microprobe consists of a 5-mm long microprobe without a tip, to which the cantilever of a second 5-mm microprobe was glued (see Figure 2). Such longer microprobes are needed to access long gas nozzles where nozzle lengths of seven times the diameter have to be measured [16].

The procedure to determine the noise was as follows. The sensor signal was recorded for a certain time and the linear drift was subtracted using a linear fit. Then, an FFT gave the power spectral density of the noise. This signal was integrated to obtain the variance and thus the noise for different bandwidths.

**Figure 3:**
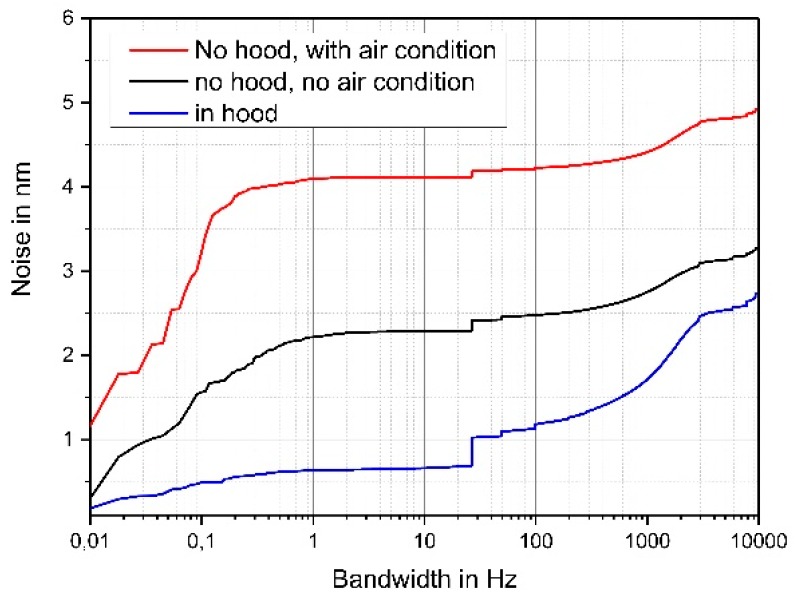
Influence of laboratory air conditioning and usage of an acoustic shielding hood on the measured noise of a 5-mm long microprobe.

First, the Profile scanner equipped with a 5-mm in length silicon microprobe was operated without an acoustic shielding hood [17]. The noise within a bandwidth of 1 Hz amounts to 4 nm (see red curve in Figure 3). Switching the laboratory air conditioning off led to a reduced noise level of approximately 2.3 nm (see black curve in Figure 3). A further reduction of the noise level was obtained by operating the Profilescanner inside an acoustic shielding box while the air conditioning was switched “on.” A noise level of approximately 0.7 nm in a bandwidth of 1 Hz was obtained (see blue curve in Figure 3).

The jump in the noise diagram in Figure 3 at 26.7 Hz is due to noise coming from a machine running in the neighboring lab. For this sensor, 50 Hz noise is very small and even in a bandwidth of 10 kHz, the noise level is less than 3 nm.

**Figure 4:**
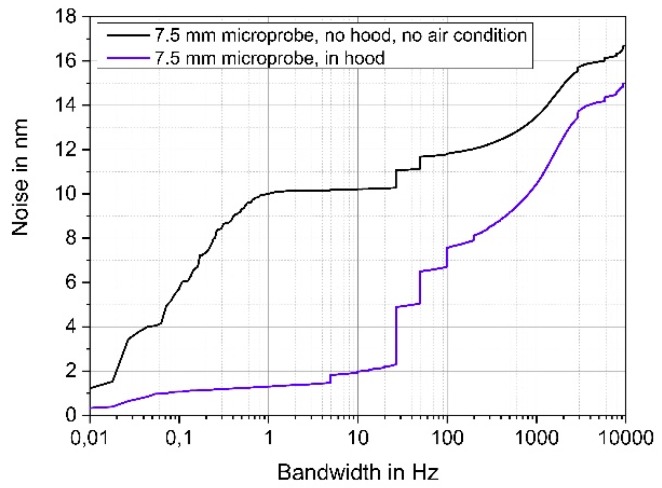
Influence of acoustic shielding hood on the measured noise of a 7.5 mm-long microprobe.

The 7.5-mm in length microprobe is much more prone to vibrations. A measurement without a shielding hood and also switching off the air conditioning in the laboratory led to a noise of 10 nm in a bandwidth of 1 Hz (see black curve in Figure 4). Operating the probe in the acoustic shielding hood reduced the noise to a level of only 1.2 nm for a bandwidth of 1 Hz. Within the 10 kHz bandwidth, the longer microprobe shows a fivefold higher noise of 15 nm compared to the 5-mm probe.

#### 2.1.3. Dynamic Noise of Microprobe for Measuring an Optical Flat

The measurements were carried out with the Profilescanner instrument (see Section 3.1) and a 5mm in length piezo-resistive microprobe with a glued-on diamond probing tip (2 μm radius and 90° tip opening angle). A commercially available data acquisition system with a DC amplifier module [18], 500X gain, and a 1 kHz low-pass filter was used for signal amplification.

**Figure 5:**
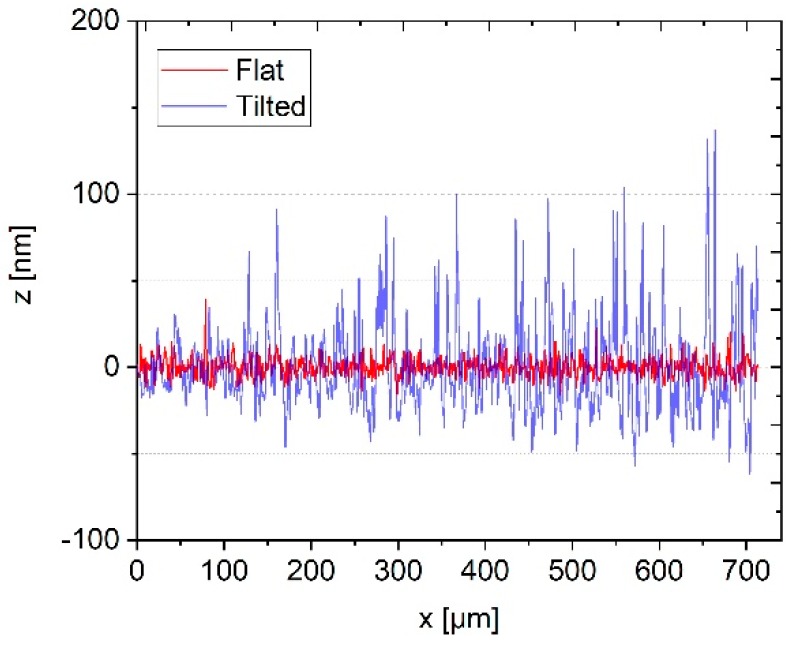
Measured roughness profiles of a well aligned optical flat (red curve) and an inclined optical flat (blue curve) for a low scanning speed of 20 µm/s.

**Figure 6:**
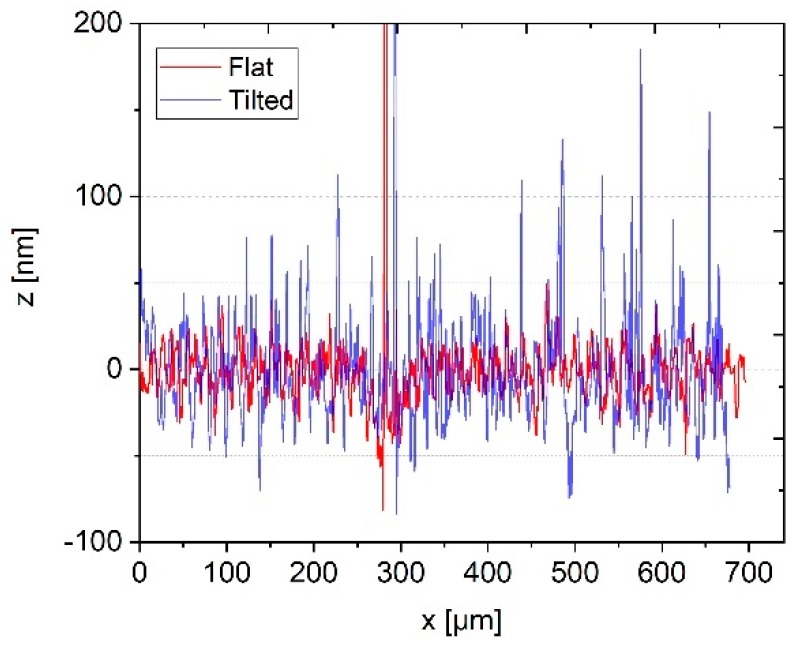
Measured roughness profiles of a well aligned optical flat (red curve) and an inclined optical flat (blue curve) for a high traverse speed of 1000 µm/s.

Profilescanner measurements were performed in the open-loop mode, i.e. with a non-controlled probing force, on an optical flat (Zerodur, flatness λ/20, serial no. 12/2, dimensions of 50 mm × 40 mm × 15 mm, manufacturer: Carl Zeiss). Measurements at four different scanning speeds were carried out on an aligned optical flat and on the same optical flat inclined by 100 µm over a measurement length of 800 µm (see Figure 5 and Figure 6).

For the measurements on the well-aligned optical flat, a microprobe pre-deflection of 8.8 µm was used to create the static probing force. Using the conversion factor of the probe deflection into the output signal of 0.057 V/μm and the microprobe stiffness of 10 N/m, a static probing force of 88 µN results. Since the measurements on the inclined optical flat leads to an increase of the probing force, a smaller pre-deflection of only 5.3 µm was used for these measurements, which correspond to a static probing force of 53 µN. 

The noise of the microprobe during measurement was evaluated using PTB’s roughness software, which is available online [19]. The waviness profile was separated from the roughness by high-pass filtering of the primary profile using a cut-off wavelength λc. Thus, the roughness profile is the difference between the primary profile and the waviness profile. In the same way, high-frequency noise was eliminated from the roughness profile by low-pass filtering with a short-wavelength filter wavelength λs. A cut-off wavelength of λc = 80 µm and a short wavelength filter of λs = 2.5 µm were used for roughness evaluation.

In Figure 5 the two measurements with and without inclination at the lowest traverse speed of 20 µm/s are compared. It can be seen that, at the first 100 µm, both measurements show comparable noise amplitudes. Through the inclination of the optical flat, the probing force is continuously increased. A measurement length of 100 µm on the inclined sample, therefore, corresponds to an increase of the probing force of approximately 125 µN. Thus, up to a probing force of 178 µN, no significant change of the microprobe noise can be observed. Beyond the 100 µm measurement length, the noise of the inclined roughness standard grows constantly. The same behavior of the microprobe can be observed for higher traverse speeds. For comparison, the measurements at 1000 µm/s are shown in Figure 6.

To quantify this behavior, we calculated the corresponding roughness parameters, according to DIN EN ISO 4287 and DIN 4768 (1990), using a bandwidth-limited phase correct profile filter, according to DIN EN ISO 16610-21 and DIN EN ISO 3274 (λs = 2.5 µm). Two roughness parameters were used for the investigations, which include the arithmetical mean roughness Ra and the maximum height Rz.

**Figure 7:**
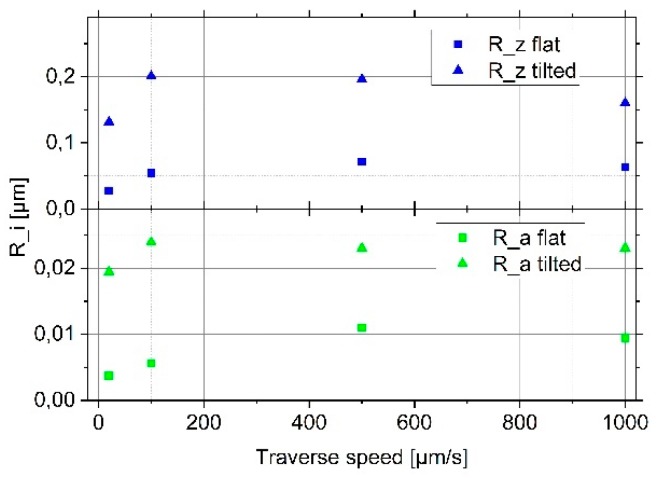
Measured roughness parameters for four different traverse speeds on a well-aligned optical flat (square symbols) and on an optical flat inclined by 100-µm to increase the probing force (triangle symbols) (Rz: blue and Ra: green).

For the lowest traverse speed of 20 µm/s, the arithmetical mean deviation increases slightly from Ra = 0.4 nm to Ra = 0.9 nm (see Table 1) when tilting the optical flat and, thereby, increasing the probing force to a value (1053 µN) higher than the recommended static probing force of 750 µN in ISO 3274. Moreover, when comparing the roughness values on an optical flat that is not tilted and on a tilted optical flat at the three higher traverse speeds (Figure 7 and Table 1), only slightly higher values are observed for the measurements on the tilted optical flat.

By comparing all four roughness values obtained for the four different traverse speeds (20/100/500/1000 µm/s), a clear linear increase can be stated for the measurements on the well-aligned optical flat. An approximate increase of the Rz and Ra values on the optical flat by a factor of 3 can be observed by increasing the traverse speed from 20 µm/s to 1000 µm/s.

Comparing the roughness values measured on the inclined optical flat and, thus, on the optical flat measured at higher static probing forces does not show a linear increase with scanning speed. The roughness values measured on the inclined optical flat at the lowest traverse speed of 20 µm/s are by a factor of five higher than those of the well-aligned optical flat are and increase to a factor of eight at a traverse speed of 100 µm/s. These values, however, nearly stay constant at 500 µm/s and 1000 µm/s. Thus, the increase of the probing force does not increase the noise level at the higher traverse speeds of 500 µm/s and 1000 µm/s.

To summarize, the increase of the traverse speed from 20 µm/s to 1000 µm/s leads to an increase of the roughness parameters Rz and Ra by a factor of three, while the increase of the probing force from 53 µN to 1053 µN leads to an increase of the roughness parameters by a factor of eight. Thus, the increase in noise is strong, but, compared with the measuring range of these sensors of some hundred micrometers, is still less than 1%. Further research is necessary at higher speeds and also more sensors have to be investigated to improve the confidence into these values.

#### 2.1.4. Dynamic Noise of Microprobe for Measuring a Roughness Standard

The same measurements as in the previous section were performed using a medium roughness standard (KNT4058/01 serial number SN6531).

Comparing the two measurements with and without inclination at the lowest traverse speed of 20 µm/s, it can be seen that both measurements show comparable noise amplitudes at the first 100 µm. Thus, for an increase of the probing force by 125 µN, no significant change of microprobe noise can be observed. Beyond a 100 µm measurement length, the measured roughness on the inclined roughness standard is larger. The same behavior of the microprobe can be observed for higher traverse speeds.

**Figure 8:**
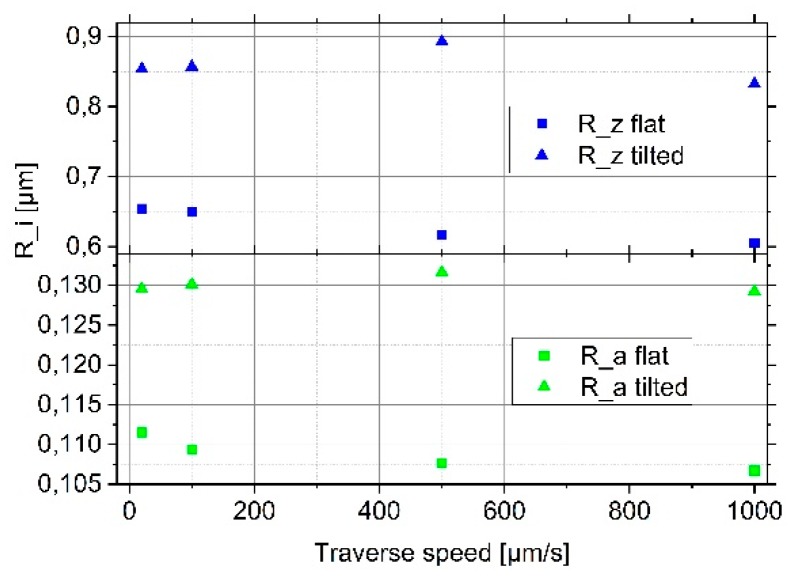
Measured roughness parameters for four different scanning speeds on a well-aligned roughness standard (RN6531) (square symbols) and on the same standard inclined by 100 µm (triangle symbols) (Rz: blue and Ra: green).

We calculated the corresponding roughness parameters again. For the lowest traverse speed of 20 µm/s, the arithmetical mean deviation increases slightly from Ra = 111 nm to Ra = 130 nm (see Figure 8 and Table 2) and the Rz value increases from 0.65 µm to 0.85 µm when tilting the roughness standard and, thereby, increasing the probing force to a value (1088 µN) higher than the recommended static probing force of 750 µN.

Increasing the traverse speed up to 1 mm/s does not lead to an increase neither of the Ra nor the Rz values. The roughness parameters stay nearly constant and seem to decrease slightly (Figure 8 and Table 2) instead of increasing with traverse speed, as expected. This behavior requires further investigation especially considering that only one sensor was examined.

### 2.2. Damping

**Figure 9:**
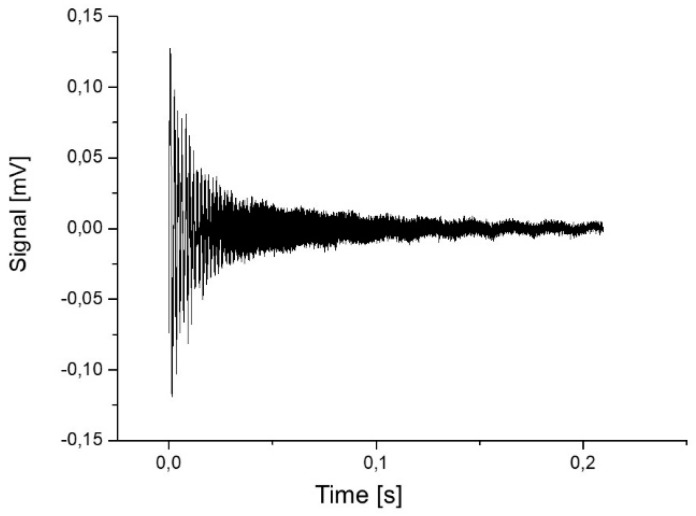
Measured microprobe output signal versus time after multi-frequent excitation of oscillations by a piezo actuator.

**Figure 10:**
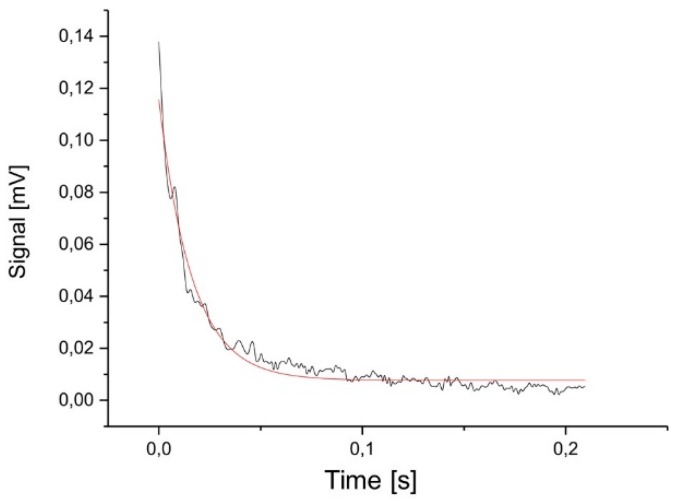
Envelope of the decaying free oscillation of a microprobe versus time (black) and fitted curve (red).

To measure the mechanical damping of one 5-mm in length microprobe, a multi-frequency excitation of the microprobe beam was created using the fast movement of a piezo actuator (see Figure 9). The oscillation amplitude created was determined (see Figure 10) and an exponential curve $A_{1}e^{\frac{t}{t_{1}}}+S_{0}$ was fitted to these data. From this fit, the decay constant *t*_1_ was determined from the half-life τ using the relationship *τ* = *t*_1_ln(2) = 0.01125 s. From a noise measurement (see Figure 3), the first resonance frequency of the microprobe was determined as 3043 Hz corresponding to an angular frequency of *ω*_0_ = 19119.7 Hz. The quality factor *Q* = 430 can be calculated from these values using the relation below.

(1) $$Q=2\omega_{0}\tau$$

From the quality factor *Q*, the damping ratio *D* can be calculated using the formula below.

(2) $$D=\frac{1}{2Q}$$

Lastly, with *D* = 1.16 × 10^−3^, the dissipation factor d = 2D can be calculated as d = 2.32 × 10^−3^. For critical damping, a damping ratio of *D* = 1, or a dissipation factor of d = 2 and a quality factor *Q* = 0.5 would be desirable. It is planned to add a viscous polymer layer to the backside of the probes to reduce the quality factor and to improve the damping ratio.

### 2.3. Tip Form Measurement

The tactile measured topography is always folded with the shape of the stylus tip. To precisely measure the width of micro and nanostructures, the influence of the stylus tip shape on the measured profile has to be corrected, according to ISO 17450, in order to determine the mechanical surface. For spherical stylus tips, morphological filter methods have been developed in ISO 16610-40 [20] and ISO 16610-41 [21].

So far, no separate filtering method exists for non-spherical probe tips, such as the pyramidal silicon tips of the micro-probes and AFM cantilevers. A prerequisite for the deconvolution of stylus tip and profile lies in the knowledge of the form of the tip. For AFM, a method for measuring the tip shape is currently being standardized in ISO 13095 [22], which is based on the determination of the sink-in depth of the tip into rectangular grooves of different widths [23]. The width of these reference structures is adapted to the AFM tip radii to be measured from 10 nm to 100 nm.

A comparable tip-testing measurement standard for the exact determination of the tip shape of the microprobes was developed. The aim was to eliminate the influence of the tip shape on the measured profiles by means of morphological filtering. This would, for the first time, enable traceable microform measurements with piezo-resistive microprobes. With its three interferometric measuring axes, the PTB’s Profilescanner (see Section 3.1) provides ideal conditions for such measurements.

The standard corresponds to a test specimen of type B1, according to DIN EN ISO 5436-1, and can also be used to determine the probe tip radius, according to DIN 32567-3: 2014-10 for spherical probes of stylus instruments [24].

**Figure 11:**
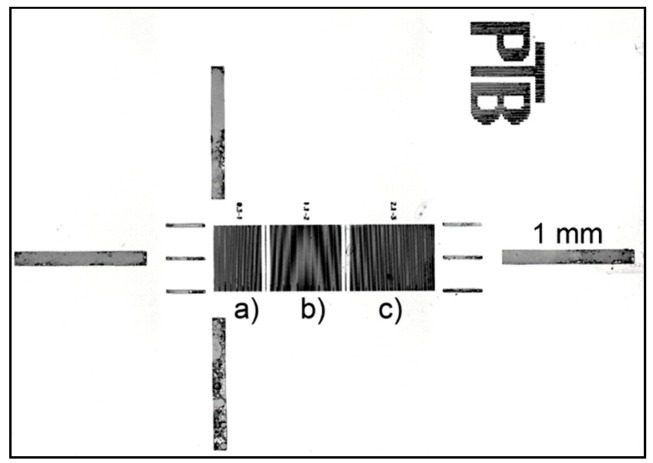
Tip-testing standard TPN 1 with three measurement areas corresponding to the different groove width ranges of a) 0.3 µm to 1.0 µm, b) 1.1 µm to 2.0 µm, and c) 2.1 µm to 3.0 µm.

The tip shape can be determined by measurements on the tip-testing standard. The standard comprises millimeter-sized finding structures and three different measurement areas with test structures in the micrometer-range (see Figure 11). The test standard is fabricated from crystalline silicon and micro-structured by anisotropic KOH-etching, which results in very precise rectangular groove structures with 90° sidewall angles and edge radii below 50 nm [9,25].

**Figure 12:**
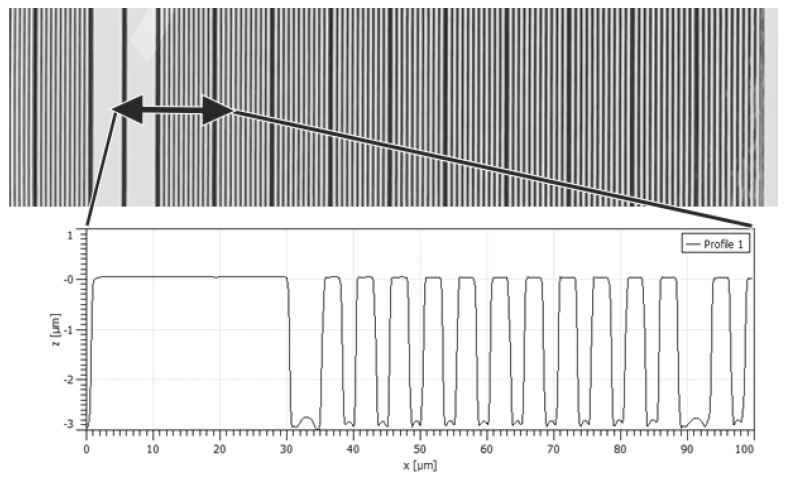
Confocal laser scanning image of the tip-testing standard showing a 30-µm wide separation structure on the left and the section of 1.1-µm in width grooves.

The three areas subdivide the tip-testing standard into three ranges of groove widths of a) 0.3 µm to 1.0 µm, b) 1.1 µm to 2.0 µm, and c) 2.1 µm to 3.0 µm. Each groove width is repeated 10 times before the next block of the next wider groove size begins. Sections of different groove widths are separated by a 10-µm wide ridge and a 5-µm wide groove separation structures (see Figure 12).

For precise form measurements of micrometer-sized objects with tactile probing tips, the shape of the tip used needs to be known to correct its influence on the measurement itself. For example, the lateral dimensions of a measured object appears to be enlarged when measured with a larger probing tip as compared to a smaller one. The diameter and the cone angle of the tip are the geometry parameters of main interest and are used in the morphological filtering routines (deconvolution). 

A first estimate of the tip diameter can be accomplished by analyzing the profile measured on the tip-testing standard for the smallest groove width with a noticeable z-depth of 3 µm. This approach could also be applied to the analysis of spherical-shaped diamond probing tips, which are commonly used in profilometers.

**Figure 13:**
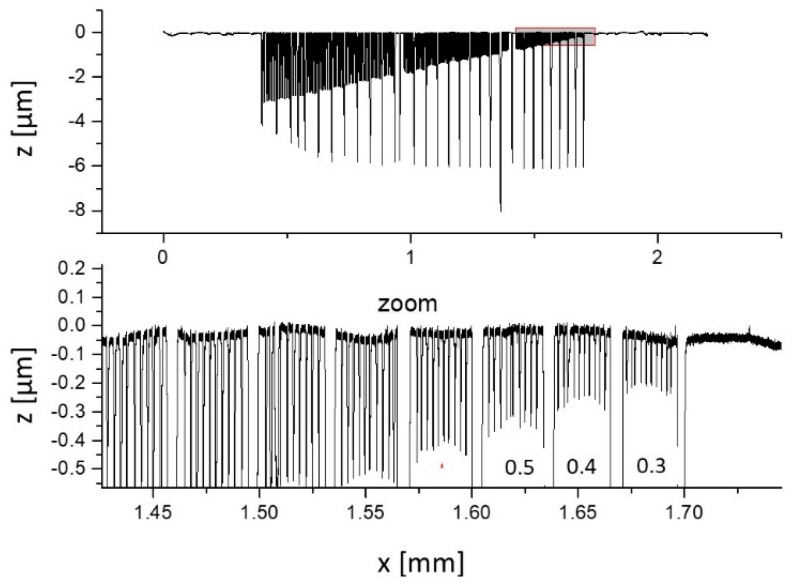
Complete profile measurement on the tip-testing standard (top) and zoomed section showing the profile for the smallest grooves (bottom).

**Figure 14:**
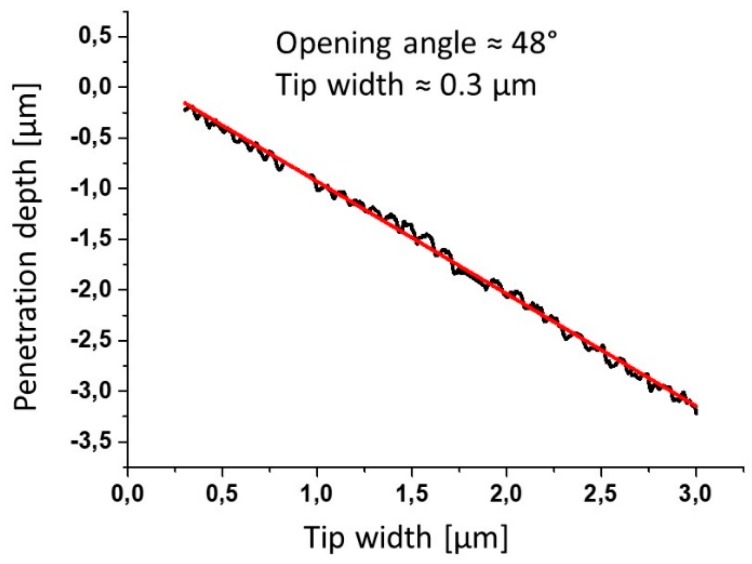
Measured sink-in depth of the tip versus groove width (see Figure 13) of the tip-testing standard. The cone angle of the tip is determined as the arctan of the slope of the linear data fit.

For non-conical (pyramidal) tips, the cone angle is calculated as the arctan to the slope of the linear fit to the data plot of measured z-depth versus the groove width (see Figure 13 and Figure 14).

**Figure 15:**
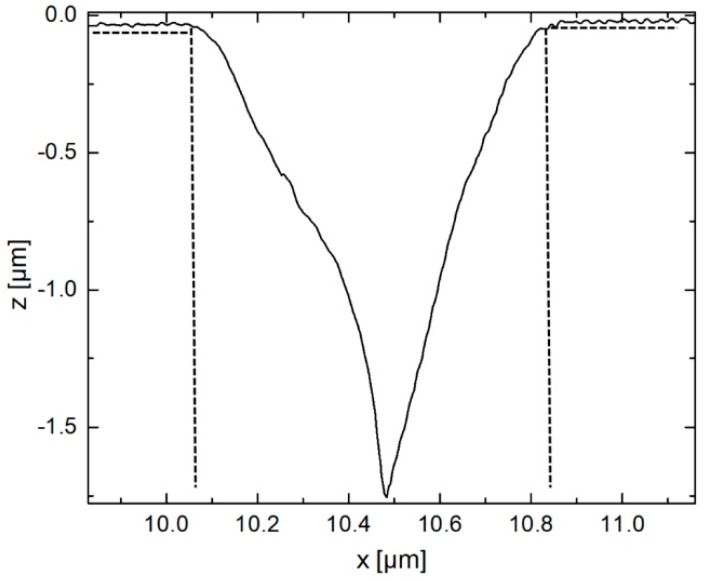
Imaging of the microprobe tip shape by measuring two successive sharp edge structures and cutting the measured profile at these points.

**Figure 16:**
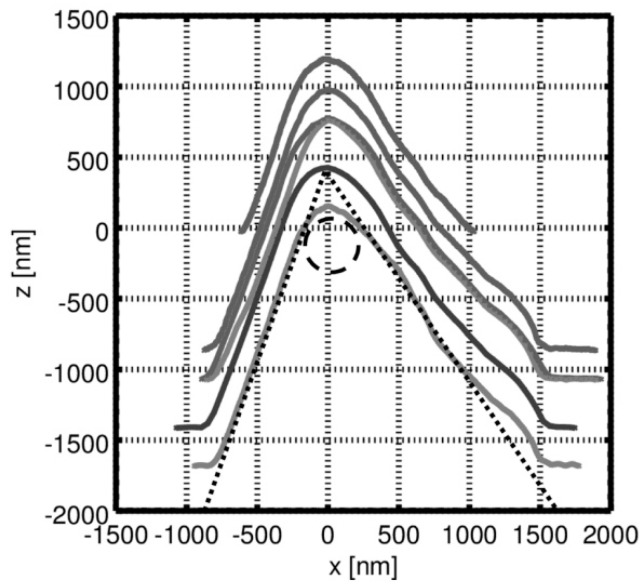
Tip shape reconstruction by matching the two previously extracted profile halves. Cone angle and diameter estimation: dashed line.

The tip-testing standard’s small edge radii (≈ 50 nm) can also be exploited as a tool for the characterization of the silicon tip. Since it is not possible to measure exactly downward along the 90° sidewall of the rectangular grooves, the shape of the tip will be the main influence on this part of the measurement profile. When the tip reaches the edge on the top ridge of the structures, the tip starts to slide down on either half of the pyramidal tip. For example, on the left edge of a groove, the right half of the pyramidal tip slides down the structure without being in contact with the sample surface on the normal contact point (tip apex). This deviation effectively leads to a measurement profile, which has the shape of the tip incorporated (see Figure 15). To extract the tip shape from the data, the profile needs to be cut at the ridge-groove-transition (the edge) and the lowest profile point (to separate the left from the right half). Afterward, the two halves are joined, which results in a representation of the tip’s form and shape (see Figure 16) that is used to determine and confirm the previously found values for the diameter and cone angle parameters.

Using this method, we were able to determine a cone angle of 52° and a tip diameter of 0.25 µm. Further research is necessary to investigate more microprobes and to compare both methods for measuring the tip shape using the new tip-testing standard.

### 2.4. Tip Wear

Roughness measurements with microprobes are feasible when using the integrated silicon tips. Tip wear is observed in dependence of traverse speed, probing force, and the hardness of the surface to be measured. Therefore, tip wear is experimentally investigated first by carrying out roughness measurements on roughness testing standards. Next, thin hard coatings of DLC and Al_2_O_3_ are tested to reduce tip wear. Lastly, the use of diamond tips, which were manually fixed to the microprobes cantilever, is presented.

#### 2.4.1. Silicon Tips

**Figure 17:**
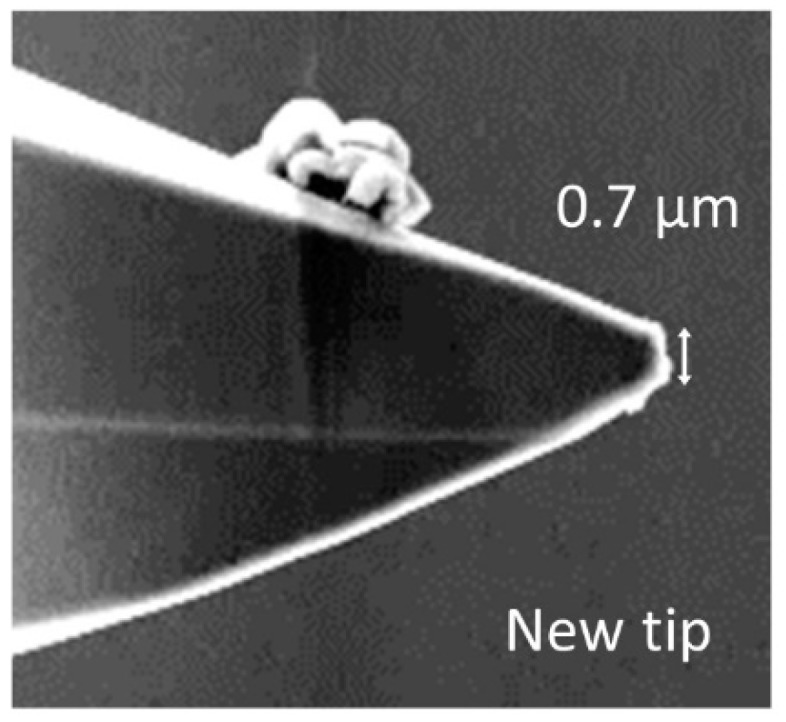
Scanning electron microscope (SEM) image of a new microprobe tip.

Measuring on hard surfaces leads to considerable wear of the integrated silicon tips. With increasing wear, the initially small tip radius (see Figure 17) increases and the lateral resolution of the microprobe decreases. For traceable roughness measurements, a spherical tip radius of 2 µm is necessary. Thus, tip wear leads to systematic deviations and wrong roughness parameters. 

Investigations at moderate traverse speeds up to 100 μm/s and very small probing forces of 6 μN, which were carried out on a roughness standard, have shown that the silicon tip wears out continuously and is flattened gradually over time. Even after a measurement length of 54 m, however, the radius of these flattened tips measured less than 1 μm and was, thus, still smaller than the radius of conventional diamond tips (2 μm). At small probing forces and moderate traverse speeds of less than 100 μm/s, reliable tactile probing and the comparability of profiles and parameters with those obtained by means of conventional procedures are possible. 

**Figure 18:**
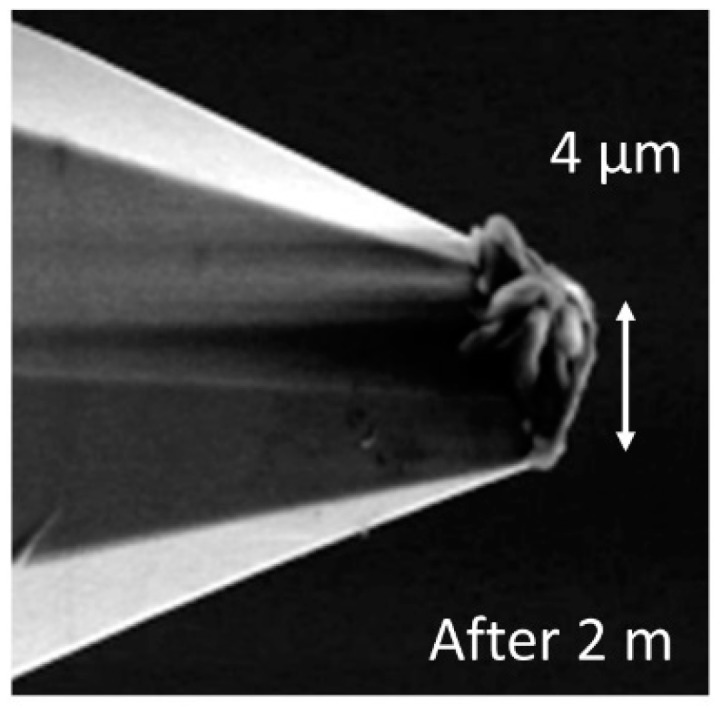
SEM image of a microprobe tip after measurements at a length of 2 meters on a roughness standard.

**Figure 19:**
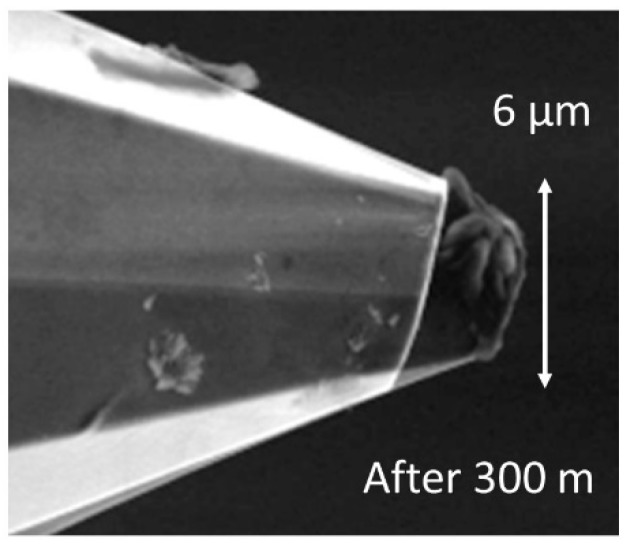
SEM image of a microprobe tip after measurements at a length of 300 meters on a roughness standard and overlaid image of the tip after 2 meters of wear length.

At the maximum possible traverse speed of these microprobes, which currently amounts to 15 mm/s [7], wear increases in such a way that, after a measurement length as short as 2 m on the roughness standard RN6531, the radius of the tip reaches more than 2 μm (see Figure 18) increasing to 3 µm after a total scan length of 300 m (Figure 19). For these measurements, we used a small static probing force of 50 µN along with a measurement length of 7 mm and, for roughness evaluation, a cut-off wavelength of 0.8 mm and software written by researchers in LabView were used.

**Figure 20:**
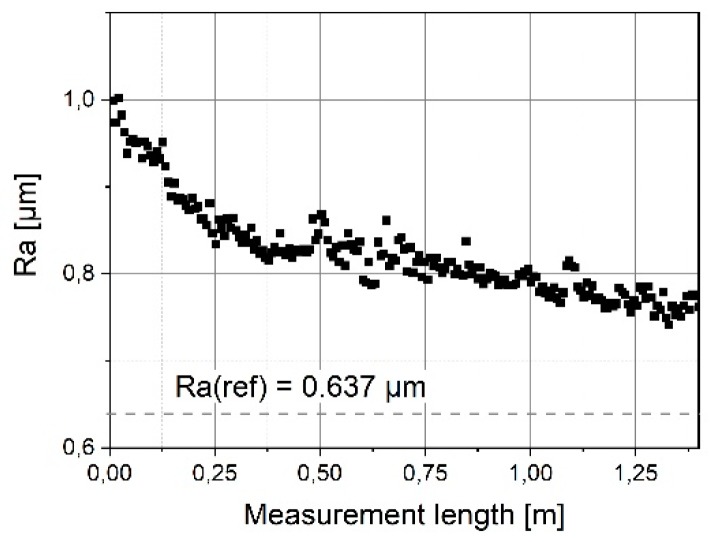
Determined average roughness values for wear measurements on the roughness standard RN6531 for a traverse length of 1.4 m. The dotted line corresponds to the reference roughness Ra = 0.637 µm.

**Figure 21:**
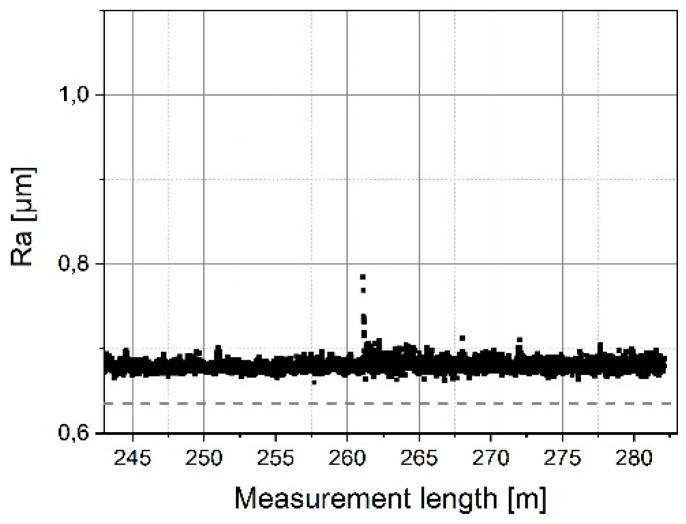
Determined average roughness values for a traverse length of 40 m after a wear measurement length of 243 m on the roughness standard RN6531.

It can be seen in Figure 20 that the initially measured Ra value of 0.9 μm decreases strongly on the first meter of the scan range. Clearly, the tip of the microprobe is still so fine at the start that larger roughness parameters than specified in the calibration certificate (Ra = 0.637 µm) are determined with the micro-probe. Subsequently, the Ra value remains stable near the calibration value of Ra = 637 nm (s. Figure 21). Thus, a mean average roughness of (650 ± 9) nm is determined over the last 21 m of the total travelling distance. This corresponds to a deviation from the reference value of only 2%.

Measuring the microprobe tip radius after 300 m of wear measurement leads to an increased value of 3 μm due to wear (see Figure 19). Thus, although roughness values might be correctly measured, the tip radius is larger than recommended for standard roughness measurements and, moreover, the form of the tip is no longer spherical, but flat and tilted due to the tilting angle of the microprobe used during wear measurements. Thus, at very high probing speeds, more resistant stylus tips are required. 

Two possible solutions to this problem, which can also be quickly implemented, include applying a hard coating to the silicon tips and gluing diamond tips to the microprobes.

##### Wear Protective Layer

Passive sensors (without piezo-resistive bridge) were used for the first wear experiments. Specimens with a wear protection layer on the tip were measured over a length of 10 m on the roughness standard. Since no electrical signal was available for the deflection of the cantilever, in this experiment, no precise statement can be made about the probing force used. It is estimated to be about 600 μN. This value significantly exceeds the value required for continuous contact. As wear protection layers of diamond-like carbon (DLC) and, alternatively, Al_2_O_3_ were tested. The DLC layer was deposited by a plasma assisted process, while the Al_2_O_3_ layer was uniformly deposited on the tip by atomic layer deposition (ALD).

Both coated tips show a similar amount of wear compared to the uncoated tip. The wear pattern suggests that the top of the probing tip breaks very quickly due to the large contact force. This means there is no more wear protection in the contact area of the tip and the tip wears in the same way than uncoated tips do. The measured wear of the tip was 2 μm on a 10-m measurement length at a probing force of 600 μN. Thus, a wear rate of 0.33 × 10^−9^/μN can be deduced. 

The microprobe provided with an Al_2_O_3_-ALD coated tip was subjected to long-term wear tests on another roughness standard (RN6678). In addition to the actual roughness measurements to create a certain wear, after each measurement on the roughness standard, a measurement on the newly developed probing tip-testing standard (see Section 2.3) was taken. This was carried out to be able to simultaneously characterize the tip wear. For these measurements, a lower probing force of 100 μN but a high traverse speed of 15 mm/s were used. The roughness parameters Ra, Rz, and Rt (total height of the roughness profile) were determined using the PTB roughness software. The contact length corresponds to the distance over which the probing tip was in contact with the roughness standard, or respectively, the probing tip-testing standard. These roughness parameters were determined not only for the measurements on the roughness standard, but also for the measurements on the probing tip-testing standard. Although the roughness parameters in itself do not provide useful information, they can, nevertheless, be employed to statistically evaluate tip wear. In addition, the tip shape can be determined directly with this standard by evaluating the penetration depth of the probing tip into the rectangular grooves of the new tip characterizer [9] (see Section 2.3).

**Figure 22:**
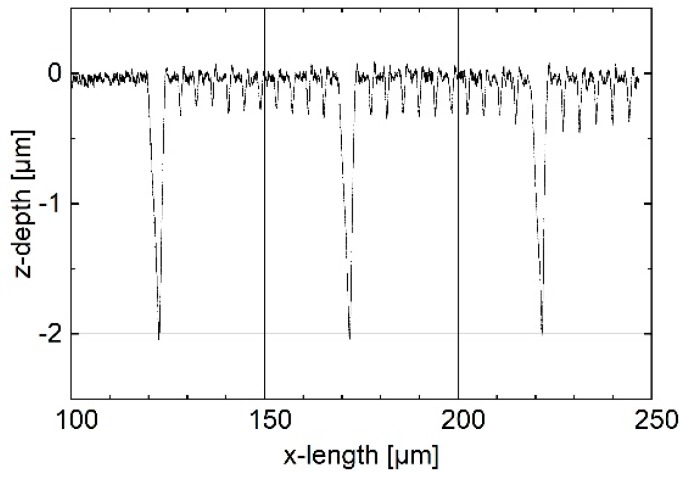
Measured profile section in the area of the grooves with a width between 1.0 μm and 1.2 μm on the probing tip-testing standard.

For the measurements on the tip-testing standard, an arbitrarily selected section of the overall profile in the area of the rectangular grooves with a width between 1 μm and 1.2 μm was selected (see Figure 22).

**Figure 23:**
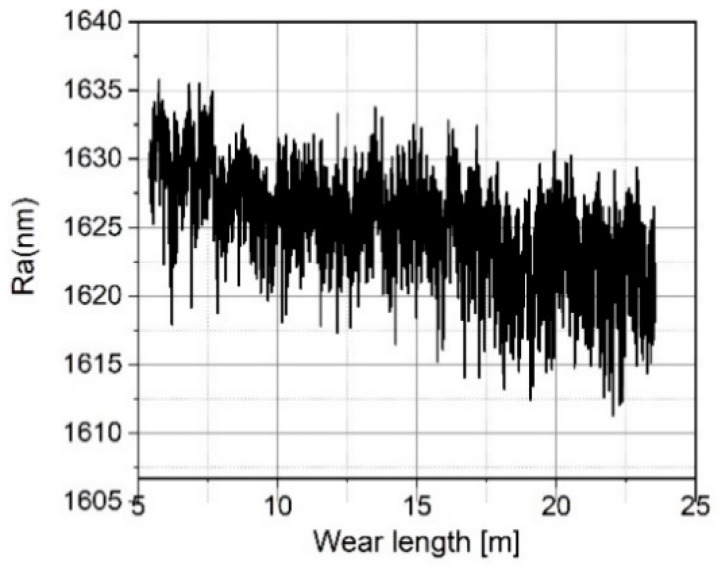
Measured arithmetic mean roughness values Ra on the roughness standard RN 6678 versus measurement length.

**Figure 24:**
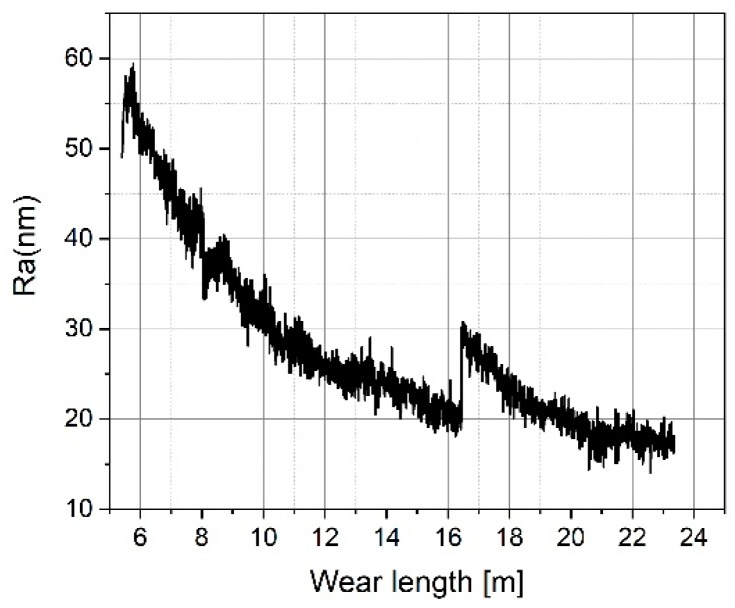
Measured arithmetic mean roughness values Ra on the tip-testing standard versus measurement length.

**Figure 25:**
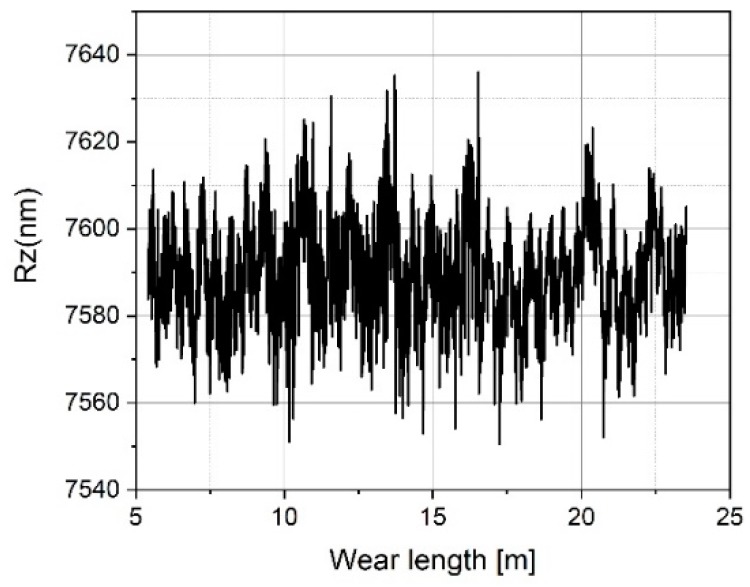
Measured Rz values on the roughness standard RN 6678 versus measurement length.

**Figure 26:**
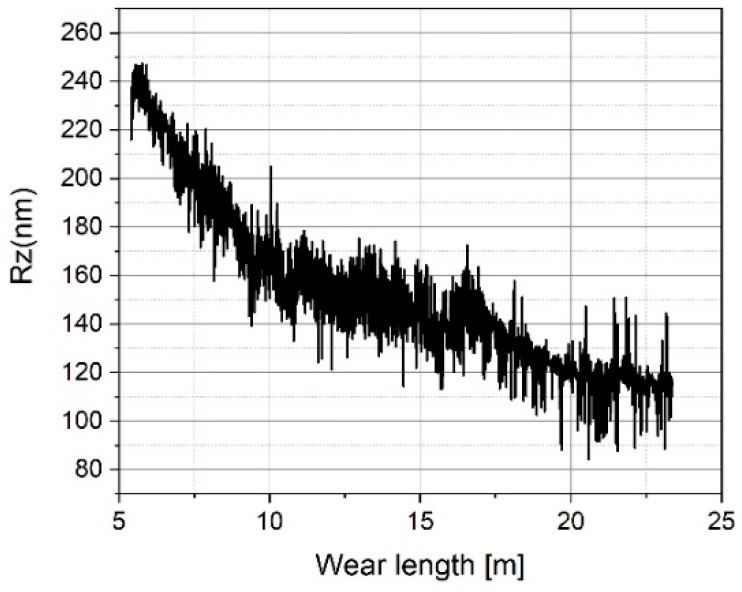
Measured Rz values on the tip-testing standard versus measurement length.

**Figure 27:**
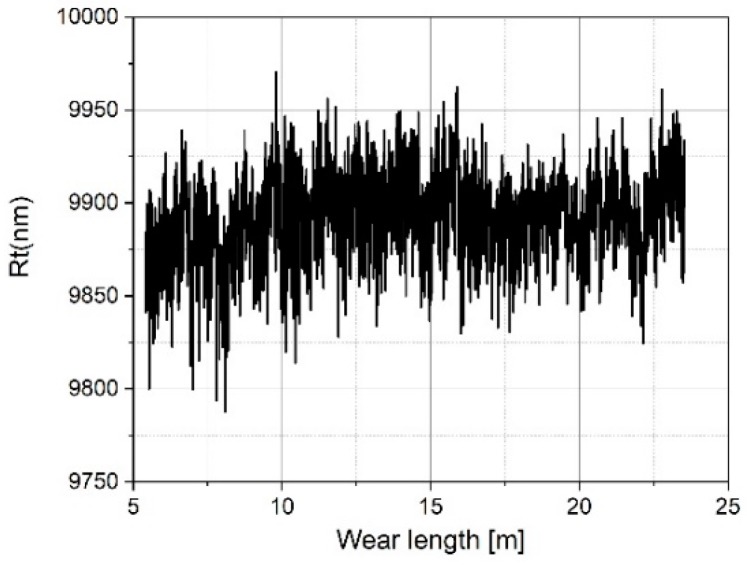
Measured Rt values on the roughness standard RN 6678 versus measurement length.

**Figure 28:**
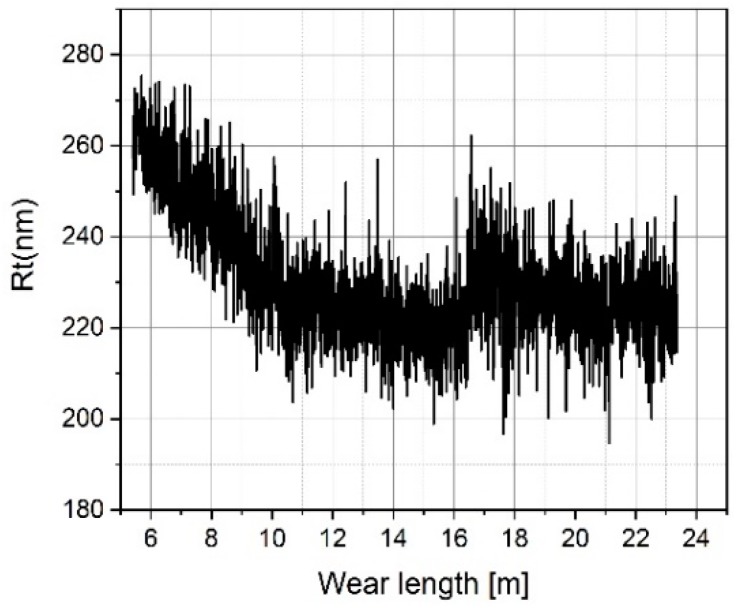
Measured Rt values on the tip-testing standard versus measurement length.

Figure 23, Figure 24, Figure 25, Figure 26, Figure 27 and Figure 28 show the roughness parameters obtained for the wear measurements on the roughness standard and in comparison with the roughness parameters obtained for the profile measurements on the tip-testing standard. The Ra-values determined on the roughness standard slightly decrease by 0.6% during 23 meters of tip wear (see Figure 23), but the Ra-value determined simultaneously on the tip testing standard strongly decreases exponentially by 69% (see Figure 24). 

After a total travelling distance of approximately 16 m, the tip-testing standard was removed and subjected to a visual inspection. Thereafter, measurements were continued with a small lateral offset, which resulted in a jump in the plot (see Figure 24).The measured mean roughness depth Rz on the roughness standard does not show a significant change (see Figure 25), but the value measured on the tip-testing standard shows a strong linear decrease up to 10 meters in wear length and then a slightly weaker decrease of 48% (see Figure 26). The two total height values of the roughness profiles Rt nearly show the same behavior as the Rz-values for the first 10 meters of wear length. There was no significant change of the value measured on the roughness standard (see Figure 27), but a linear decrease of the Rt-value at the first 10 meters. Then, for the remaining 13 m of wear length, no significant change of the Rt-value can be observed (see Figure 28). 

These measurements show that the new tip-testing standard with rectangular grooves of widths below the recommended tip radius of 2 µm for standard roughness measurements offers a simple and powerful means to determine tip wear.

Another evaluation method is to monitor the penetration depth into the rectangular grooves on the tip-testing standard [7]. By evaluating the trend of the penetration depth over the abrasion length, the tip wear can be easily determined. The strongest tip wear was observed during the first 300 mm of abrasion length. The penetration change amounts to 0.3 µm, which leads to an abrasion slope of 10^−8^/µN. During the next 400 mm of abrasion length, a much smaller slope of 2.5 × 10^−9^/µN was observed (for more details, see [7]).

The roughness parameters measured on the roughness standard RN6678 barely vary, as already observed for uncoated tips, even though the silicon tips continuously wear out. The measurements with the help of the newly developed probing-tip reference standard allow the tracking and measuring of this continuous wear. In conclusion, it can be stated that the goal of reducing silicon microprobe tip wear due to thin wear-resistant coatings has not been achieved and further research is necessary to develop more wear resistant microprobe tips.

#### 2.4.2. Diamond Tips

**Figure 29:**
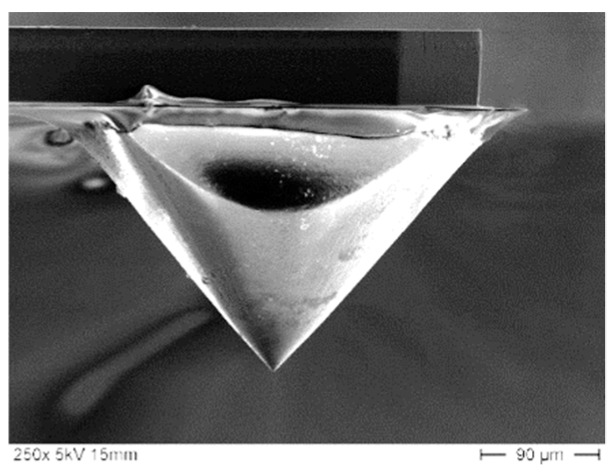
Piezo-resistive microprobe with a spherical 2 µm radius diamond tip glued onto the free end of the cantilever.

**Figure 30:**
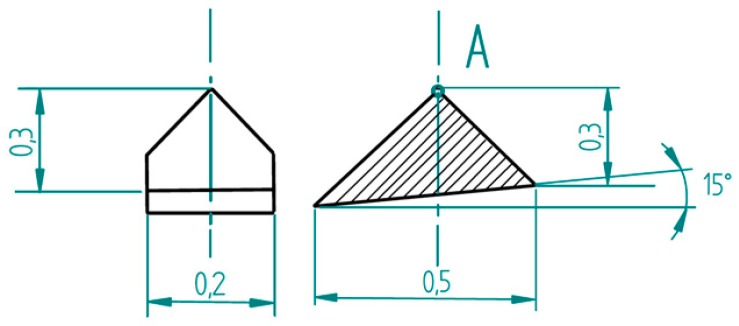
Diamond probing tips with 2 μm radius and 90° opening angle.

Commercially available conical diamond tips with a 90° opening angle and a spherical 2 µm radius were glued onto “tip-less” microprobes (see Figure 29) using a two-component epoxy resin adhesive [26]. Care had to be taken not to use a viscous glue, since this would have led to unwanted drift phenomena during measurements. The diamonds were cut under an angle of 15° since these microprobes were also used with an inclination of 15° (see Figure 30).

**Figure 31:**
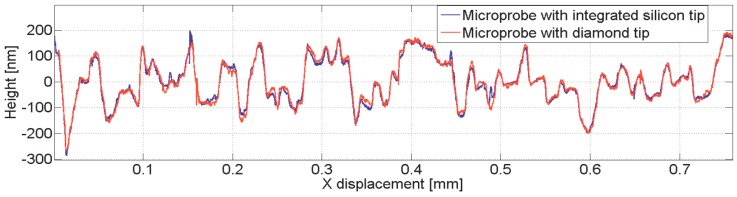
Comparison measurement of a super fine roughness standard between a microprobe with silicon tip and a microprobe with a diamond tip.

Various investigations were performed using the Profilescanner to inspect the characteristics of the microprobe with the mounted diamond tip. Comparison measurements between diamond-tip microprobes and integrated silicon-tip microprobes confirm the feasibility of this solution [16] (see Figure 31).

Within the scope of MicroProbes [8], an EU EMPIR project, longer pencil shaped diamond tips were developed and are currently being prepared for the first test measurements.

### 2.5. Probing Force

To prevent tactile measuring systems at high traverse speeds from tip flight, the probing force is usually increased. This may lead to surface scratching and, therefore, the tip radius used usually has to be increased, which, in turn, leads to a loss of lateral resolution. Using microprobes with silicon tips, with typical radii of unused tips of approximately 50 nm, leads to the necessity to limit the traverse speed in order to not scratch the surface to be measured. 

Compared to the probing force of 750 μN recommended for tactile roughness measurements with a 2-µm tip radius, the probing force required to prevent the probe from lifting off from the surface due to dynamic forces during a measurement at 15 mm/s traverse speed was calculated to be only 28 μN for the 5-mm long microprobes [7]. 

Although high traverse speeds up to 15 mm/s have been tested for the long slender microprobes, the maximum scanning speed for reliable and non-destructive measurements needs to be determined. Future systematic investigations on tip flight and on surface scratching are necessary to precisely model the behavior of the long slender microprobes and to calculate the maximum scanning speed with dependence on surface hardness and to set the necessary probing force to prevent tip flight and surface scratching. 

Microprobes can be operated in two measurement modes including open and closed loop for the probing force. The closed loop mode allows us to measure roughness at constant small probing forces down to 1 µN. The advantage is that the inclination of the probe does not change during the measurement and morphological filtering is relatively easy. 

Open-loop measurements without controlling the microprobe deflection allows much faster roughness measurements, since no servo loop with limited bandwidth has to be used. In this mode, the probing force changes with the measured height, or with the deflection of the microprobe cantilever. Therefore, high probing forces may occur during measurements. 

To exactly measure the microprobe probing force, different methods exist. The most frequently used methods use reference springs with a known stiffness [27,28], high-precision compensation balances [29], and well-calibrated force sensors [30,31,32].

The German DIN standard 32567-3 describes in detail the usage of reference springs for the force calibration of stylus instruments [24].

Using force sensors for the force calibration of microprobes is much easier than using reference springs, but the size of the probing area is a problem. It is quite small (50 µm by 50 µm) for some sensors. The expanded uncertainty of this type of force sensor can reach values as low as 0.24% [33]. A few years ago, a new force sensor specially developed for stylus instruments offering a large loading area of 0.7 mm × 1 mm was developed [32]. Thus, the calibration of probing forces of the developed long microprobes seems to be possible at uncertainty levels of 1%. 

## 3. Currently Available Instruments Equipped with Piezo-Resistive Microprobes

Currently, microprobes are being used in two experimental set-ups. On one instrument developed by PTB researchers known as the Profilescanner and moreover, on a miniaturized Micro Profiler with an integrated feed unit for traverse speeds up to 5 mm/s specifically developed for the measurements in deep bores.

### 3.1. A Traceable Profilescanner for Measurements inside High-Aspect Ratio Microstructures

PTB, in cooperation with the CiS Forschungsinstitut für Mikrosensorik GmbH in Erfurt and the Institut für Halbleitertechnik (Institute of Semiconductor Technology – IHT) of Braunschweig Technical University, has developed a profilometer for roughness measurements on the surfaces of micro-components with a high aspect ratio [34]. Its key component is the long silicon microprobe. The microprobes are available in different lengths (1.25 mm, 3 mm, and 5 mm) and widths (30 μm, 100 μm, and 200 μm) and with tip heights of up to 100 μm. The sensor is mounted into a measuring head with a travelling distance of 800 μm × 800 μm × 250 μm. Three laser interferometers with a resolution of 1.24 nm are also integrated. They measure the position of the tip virtually. A motorized 3D moving stage allows the precise positioning of samples. 

The Profilescanner is operated within an acoustic isolation chamber in a laboratory that is temperature-controlled and humidity-controlled. External vibrations are reduced through a passive anti-vibration platform. The measurement uncertainty mainly comprises four parts: cantilever noise and cantilever sensitivity uncertainty, residual Abbe error, laser interferometers, and environmental influences like thermal drift. Numerous comparison measurements of step heights and of PTB roughness standards proved that the deviations of the profilometer for step heights as well as for roughness were within ±10 nm [34]. The expanded uncertainty for roughness and step height measurements is estimated at 5.2 nm (k = 2) for a 1-µm step height or a roughness value [34]. Table 3 shows the specifications of the Profilescanner.

**Figure 32:**
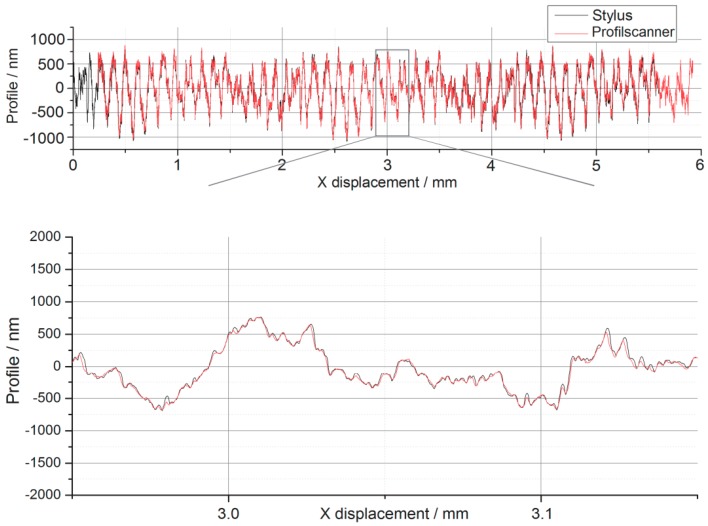
Comparison of stitched roughness measurement of the Profilescanner (red) and a roughness measurement nearly at the same position with PTB’s traceable roughness measuring stylus instrument (black).

Since the maximum measurement range of the Profilescanner is limited to 800 μm, a stitching procedure using cross correlation was developed to combine several successive profiles into one common profile (see Figure 32). Although the reference stylus instrument and the Profilescanner did not measure at exactly the same position on the roughness standard, a very good comparison of both profiles was obtained.

### 3.2. A Miniaturized MicroProfiler with an Integrated Feed Unit

**Figure 33:**
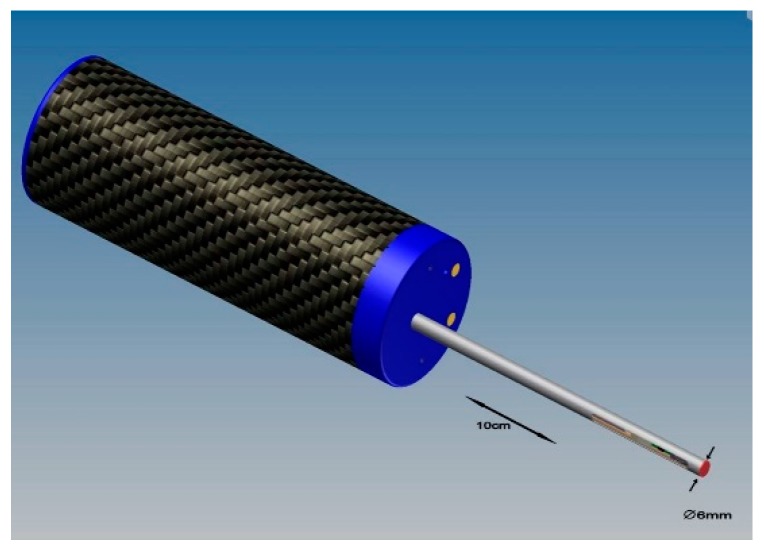
MicroProfiler-B with integrated feed-unit for up to 5 mm/s scanning speed inside deep bores.

**Figure 34:**
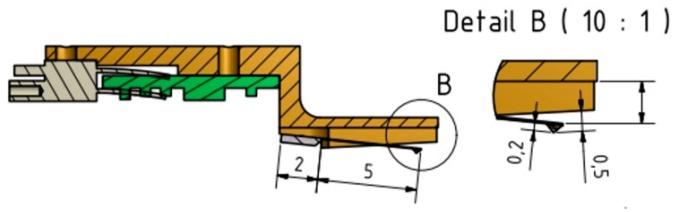
Cross section through the measuring head of a MicroProfiler-B with the inclined cantilever microprobe.

Recently, a miniature roughness measurement system for bores, the MicroProfiler-B, was developed (see Figure 33) [35]. The battery-operated hand-held miniature roughness tester features Bluetooth data transmission to a computer and a very short measurement loop due to an integrated skid. It was specifically designed for fast and easy roughness measurements, especially in very deep bores. The cantilever-based measurement system has a volume of a few mm^3^ only and is mounted inside a skid body (see Figure 34). The measurement data is generated and amplified very close to the stylus. The stylus is lowered only when the skid has touched the surface and, therefore, the gauge is easy to handle. The instrument serves to measure and calculate the roughness parameters, according to the valid ISO standards. The new miniature pick-up design enables much higher scanning speeds than conventional instruments. Profile lengths of up to 12.5 mm at traverse speeds between 0.5 mm/s and 5 mm/s can be measured. Calibration checks can be carried out in pre-programmed intervals. For bore diameters larger than 6 mm, suitable adapter pieces are mounted onto the guiding pipe. The stylus has open access to the work piece surface only during the measurement (see Figure 34). Otherwise, the 100-mm long guiding pipe is closed. The vertical measurement range amounts to 100 µm at a resolution of 18 Bit. The stylus diamond tip radius is 2 µm and the stylus cone angle is 90°.

**Figure 35:**
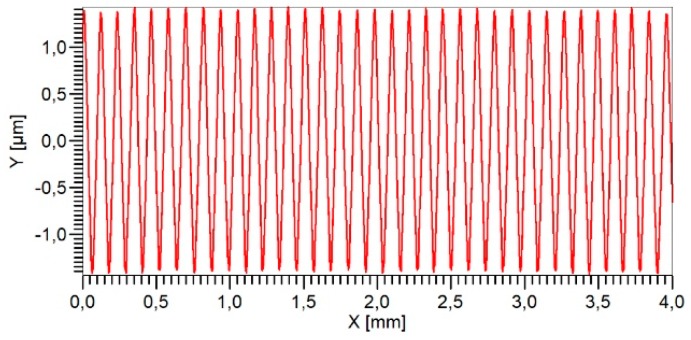
Micro Profiler measurement on a sinusoidal roughness artefact.

**Figure 36:**
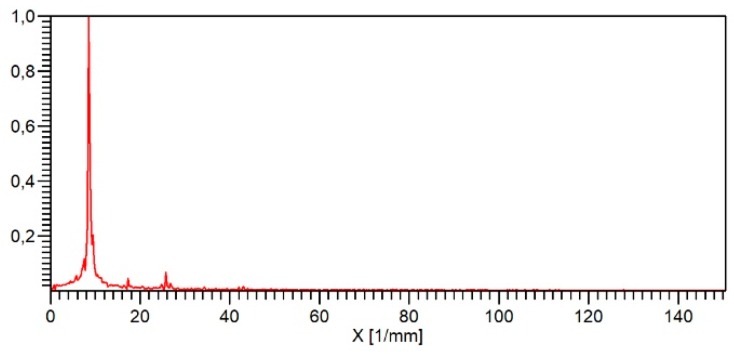
FFT spectrum of the profile measured on the sinusoidal artefact (see Figure 35).

To test the Micro Profiler, a sinusoidal roughness artefact [36] with a height of 3 µm and a lateral period of 120 µm made of glass was measured at a traverse speed of 2 mm/s (Figure 35). The measured amplitudes are very uniform and the Fast Fourier Transformation (FFT) spectrum of the profile (Figure 36) also shows very small higher harmonics of the fundamental frequency. The amplitudes of the second harmonic at 16.7 mm^−1^ amounts to 4% and that of the third harmonic to only 7%. Further research is necessary to fully characterize this new microprobe with an integrated feed unit.

## 4. Experimental Results

Up to now, the main application of these slender microprobes has been for fast roughness and form measurements inside micro-nozzles, like critical nozzles for gas flow measurements and diesel injection nozzles. Within the EU MicroProbes project [8], these microprobes have been attached to manufacturing machines and a new application has been added including the measurement of mechanical parameters of surfaces.

### 4.1. Measurement of Roughness and Form Deviation inside Micro-Nozzles

The microprobes with a total length of 1.25 mm were specifically designed for the measurement of micro-nozzles. The slender cantilever has a width of 30 µm and also a small total height of < 50 µm (cantilever height plus tip height). Thus, it is able to measure roughness and form in micro-nozzles with diameters down to approximately 70 µm. Measurements on gas and injection nozzles are presented as examples.

#### 4.1.1. Critical Flow Venturi Nozzles (Sonic Nozzles) for Gas Flow Measurements

Due to their long-term accuracy and excellent repeatability, sonic nozzles have been used in a number of industrial and scientific applications for gas and fluid flow measurements, such as calibration standards. In the case of sonic nozzles, the flow rate remains constant over the nozzle when the differential pressure exceeds a certain minimum value. The smaller the flow rate, the smaller the diameter of the corresponding nozzle must be. 

With smaller nozzles, however, the topography of the inner surface has an increased influence on the flow behavior and the flow rate. For nozzles with a diameter below 1.5 mm, significant deviations from the specified ideal shape and surface may occur. These deviations can turn out to be extremely disturbing in practice and we do not yet understand why they occur in connection with the inner topography. Measuring the inner surface of such small nozzles with conventional tactile and optical measuring instruments is, however, very difficult.

**Figure 37:**
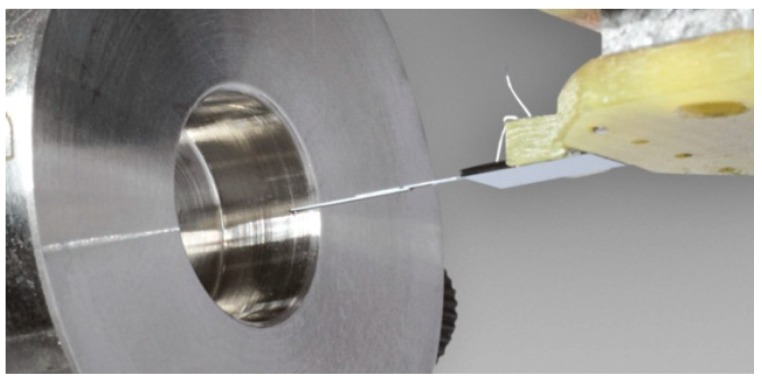
The microprobe in front of a nozzle with 800 μm diameter. The nozzle opening itself is not visible. The cantilever is 7.5 mm long. It consists of two cantilevers that are glued together.

Diverse nozzles manufactured by different methods (e.g. turning or electrical discharge machining) have been measured by the Profilescanner. Their diameters varied from 200 μm to 800 μm and the measuring length ranged from 1.5 mm to 7 mm (see Figure 37). It is the first time that the inner surface of sonic nozzles with diameters in the micrometer range has been successfully characterized [16,37].

Manufacturing defects were detected in some nozzles, which explained the deviation of the flow rate experiments performed earlier. This also confirms the assumption that the shape and the roughness of the inner surfaces have a strong influence on the flow rate properties of the nozzles [38]. It is expected that the calibration uncertainty and the quality control of such nozzles with diameters in the micrometer range can be improved by the measurement results of the Profilescanner.

#### 4.1.2. Diesel Injection Nozzles

The slender piezo-resistive microprobes were used for the nondestructive quality testing of high-aspect ratio injector nozzles of diesel engines [3]. The form and roughness directly inside the injector nozzle holes were measured. A comprehensive study with the current generations of state-of-the art injector nozzles as well as prototype holes was carried out. The potential of the silicon microprobe for a systematic improvement of micro bores was demonstrated with bores of 100–200 μm in diameter fabricated using electro discharge machining. The results were validated in comparison with the standard roughness measurement method after cutting the nozzles into two halves.

For the measurements, slender silicon cantilevers with a length *l* = 1.25 mm, a width *w* = 30 μm, and a thickness *h* = 25 μm [15] were employed. A Wheatstone bridge supply voltage *V*_B_ = 1 V was used for the measurements. The silicon tip consists of a micro pyramid with an octagonal base with a height between 15 and 25 µm, an angle of apex of approximately 40°, and initial radii down to 100 nm (see Table 4). The tip sidewalls are determined by the emerging {311} silicon crystal facets.

**Figure 38:**
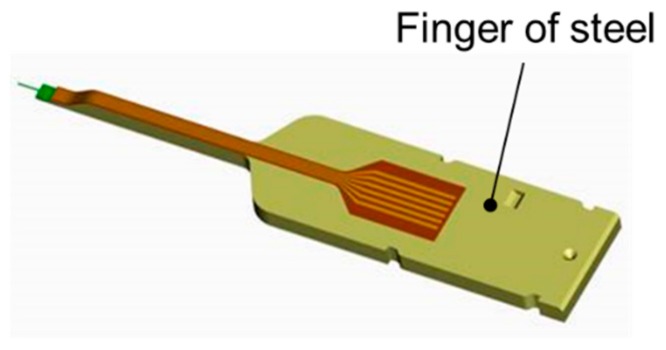
Microprobe on steel finger carrier developed in the course of µgeoMess, a BMBF funded project [13].

The microprobe was mounted in a customer-specified package comprising a finger-shaped steel substrate, on which the sensor was glued and a plastic housing designed to protect the cantilever when the sensor is off [13,39] (see Figure 38).

**Figure 39:**
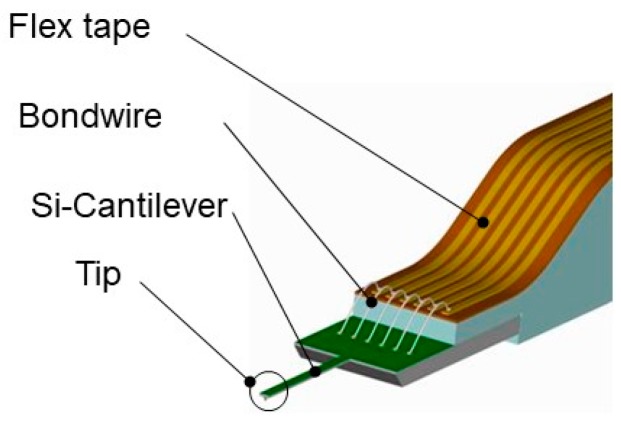
Detailed view of Si-cantilever microprobe, bonding wires, and flex tape for electrical connection [13].

The microprobe was attached to the Profilescanner instrument. The power supply to the strain gauge and the signal read-out were realized by gold wire bonding to a flexible printed circuit board (FPCB), which was glued onto the steel substrate (see Figure 39). The sensor was connected to a DC instrumentation amplifier in a four-wire technique [18]. The specifications of these sensor prototypes are shown in Table 4.

**Figure 40:**
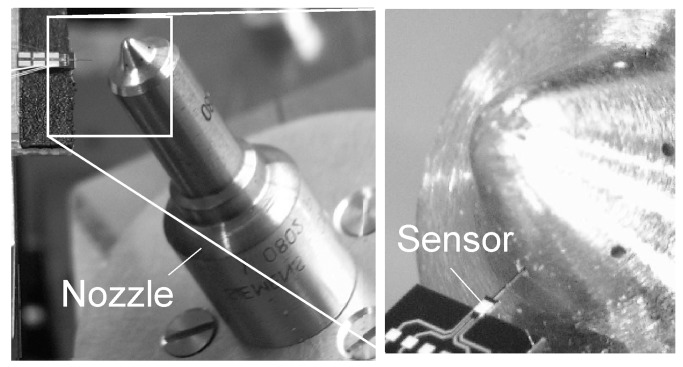
A prototype valve controlled orifice (VCO) nozzle of a diesel engine and the microprobe entering one of the spray holes for surface quality measurement.

For internal geometry measurements, the nozzles under test were arranged on a rotating/tilting stage (see Figure 40). Temperature fluctuations affecting the sensor response typically by ∼ 10 nm/°C could be neglected due to the short measurement duration of typically only 1 to 20 s.

Prior to the surface topography measurements, the noise behavior of the sensor prototypes was measured to be 4 nm in a bandwidth of 1.6 kHz [3]. Next, the repeatability of the measured surface profiles was determined by repeating the measured profiles two times. The three profiles matched within ±2 nm, which indicates the excellent repeatability obtained. Roughness parameters were calculated using *λc* = 0.25 mm [5]. Average values of *Ra* = 419 nm and *Rz* = 2.48 μm with maximum deviations of less than 5 nm and 0.02 μm, respectively, were obtained for the three repeated profiles.

The influence of the sampling rate and the probing force on tactile roughness measurements was investigated. Scans were performed at various probing forces and various sampling distances on roughness artifacts. A scan length of 0.9 mm was selected. Reliable measurements and uncertainties of *Ra* and *Rz* around 10 nm and 0.2 μm, respectively, were found at data point densities of > 1/μm and probing forces of > 200 µN.

With these measurement parameters, measurements were undertaken at micro holes of valve controlled orifice (VCO) nozzles and micro-sac injector nozzles, which were both fabricated by electrical-discharge machining (EDM). Seven holes per nozzle were measured. A reduction of the roughness parameters to *Ra* ≈ 400 nm and *Rz* ≈ 2.5 μm with the hole diameter decreasing to ≤ 140 μm was observed. 

To check the potential of the sensor in the course of a further reduction of the hole size, holes of 100 μm in diameter on steel plates of 0.7 mm thickness were also characterized. Flat profiles without parabolic deviations were measured, which indicates that the holes were accurately scanned along their crown line. Measured roughness parameters *Ra* = (570 ± 50) nm and *Rz* = (3.9 ± 0.4) μm were compared with conventional stylus instrument measurements at sectioned samples. A very good agreement was found for *Ra* = 563 nm and also for *Rz* = 2.5 μm. Due to its larger tip radius, the stylus instrument seemed unable to access the deep craters on the surface of the nozzle. Therefore, the differences in surface finish of the investigated holes can be more clearly seen using the piezo-resistive microprobe. The tip size, angle, and radius of the microprobe are better matched to the micro hole form and roughness compared with standard stylus instruments [3].

**Figure 41:**
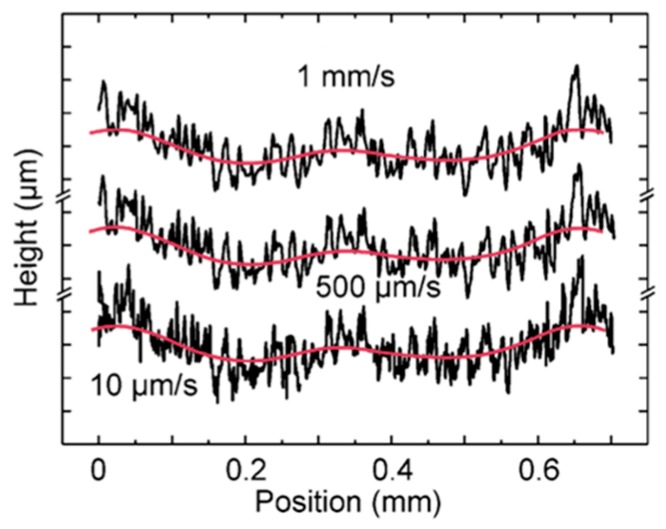
Roughness measurements inside a micro sac nozzle at three different traverse speeds.

Due to its small mass, high stiffness, and large resonance frequency, high scanning speeds in a range of 50 μm/s to 1 mm/s (see Figure 41) without interfering stick-slip or inertia effects could, for the first time, be demonstrated.

### 4.2. Mechanical Properties Measurement via Contact Resonance 

The mechanical properties of micro-structures and nanostructures can be measured by nano-indentation or AFM. AFM measurements permit topography measurements and simultaneous measurements of surface stiffness [40,41]. However, the AFM cantilever is only several hundred µm long and sometimes not long enough to measure inside micro-structures. Therefore, the application of the contact resonance method to the slender piezo-resistive cantilever was tested. 

The technique analyzes the resonance frequency changes of the cantilever in elastic contact to a surface. The indentation modulus of the sample surface can be determined by measuring these frequency shifts [42]. 

In the contact resonance method, it is most important to model the cantilever flexural vibrations and the tip-sample interaction appropriately. The point-mass model is often used to calculate the first free flexural resonance frequency *ω*_0_ and the contact resonance frequency *ω* of the cantilever.

(3) $$\omega_{0}=\sqrt{\frac{k_{C}}{m^{*}}}$$

(4) $$\omega =\omega_{0}\sqrt{1+\frac{k^{*}}{k_{C}}}$$

where *k_C_* and m* are the static spring constant and effective mass of the cantilever and k* is the normal contact stiffness.

The tip-sample interaction is often modelled as that of a spherical tip with radius R contacting a flat sample surface with a normal force *F_n_*, according to the Hertzian model for elastic deformation. 

The normal contact stiffness k* is then given by the formula below.

(5) $$k^{*}=\sqrt[{3}]{6E^{*2}RF_{n}}$$

where E* is the reduced Young’s modulus of contact, which is given by the equation below.

(6) $$\frac{1}{E^{*}}=\frac{1}{M_{s}}+\frac{1}{M_{t}}$$

In this case, *M_s_* and *M_t_* are the indentation moduli of the sample and the tip, respectively. 

**Figure 42:**
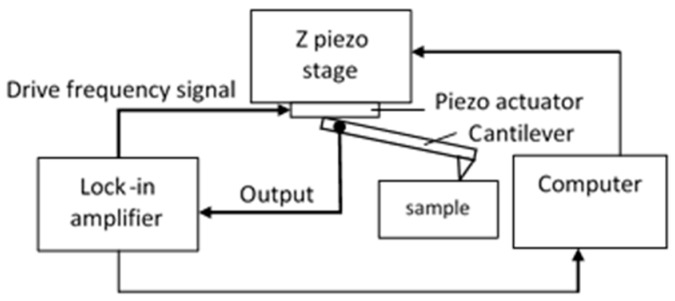
The contact resonance experimental setup realized on the Profilescanner.

The contact resonance technique is realized on the Profilescanner in the ultrasonic atomic force microscope (UAFM) mode [43]. This means that the cantilever, instead of the sample, vibrates. The cantilever is fixed on a *z*-piezo stage (PI, model P-622.ZCD) and the base of the cantilever is excited by a piezo actuator. A lock-in amplifier (SRS, model: SR830) excites the piezo actuator and analyzes the spectrum of the cantilever vibrations (see Figure 42).

A 5-mm long microprobe was used in the experiments. The static spring constant of the cantilever was calibrated to be *k_C_* = 13.2 N/m ± 4%. Reference measurements were used in the experiment to precisely determine the form and radius of the cantilever tip. A polycarbonate standard sample worked as a reference material to determine the elastic modulus of a stripe-like specimen made of SU-8 on a silicon wafer. The evaluation method using a reference material is described in more detail in [44]. Polycarbonate was chosen as a reference because its indentation modulus is similar to that of the SU-8 material to be measured. SU-8 was chosen as a test material because it has a low indentation modulus. A low indentation modulus is necessary because the force measurement range of the microprobes is limited due to their low stiffness.

The indentation modulus of the polycarbonate standard sample was 2.7 GPa and the reduced modulus with a diamond probe was 3.10 GPa. The indentation modulus of the silicon cantilever tip is assumed to be 165 GPa.

**Figure 43:**
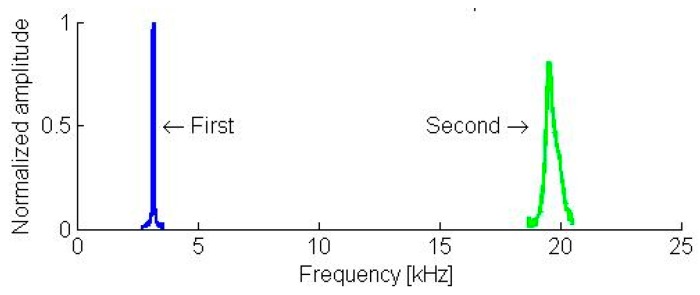
The free vibration resonance spectrum of a 5 mm long piezo-resistive cantilever microprobe.

**Figure 44:**
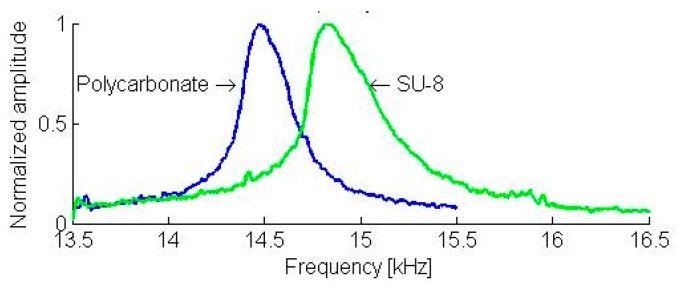
The contact resonance spectra of the first flexural mode of a 5-mm in length piezo-resistive cantilever microprobe in mechanical contact with a polycarbonate (blue curve) and an SU-8 sample.

**Figure 45:**
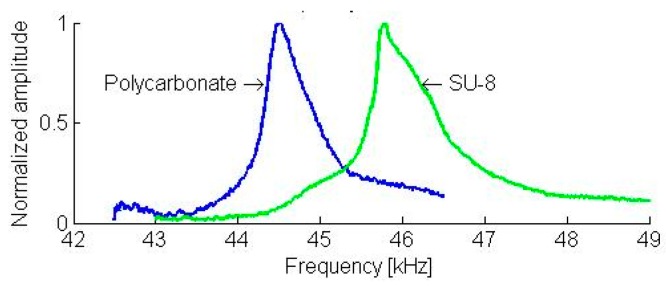
The contact resonance spectra of the second flexural mode of a 5-mm long piezo-resistive cantilever microprobe in mechanical contact with a polycarbonate (blue curve) and an SU-8 sample.

The resonant frequencies of the first two flexural modes are 3.1 kHz and 19.6 kHz, respectively (see Figure 43). The static load of the cantilever on the sample was 132 µN. On the polycarbonate and SU-8 samples, the contact resonance frequencies of the first two flexural modes shifted to 14.5 kHz, 14.8 kHz (see Figure 44), 44.5 kHz, and 45.8 kHz (see Figure 45), respectively. Applying the measured frequencies to the Equations (3)–(6), the indentation modulus of the photoresist SU-8 is calculated as being 3.41 GPa. This result is clearly smaller (−25%) than the evaluated value of 4.50 GPa using nano-indentation and the literature value (i.e., $\frac{E}{1-v_{SU-8}^{2}}=4.25\pm 0.73$ GPa, where *ν*_*SU*-8_ = 0.22) [45].

The experimental results indicate that the utilization of the contact resonance method on the piezo-resistive cantilever is possible. To improve the precision of the method, the modelling and the theoretical analysis must be improved.

## 5. Summary

Long, slender silicon microprobes with piezo-resistive sensing of the cantilever deflection are commercially available and can be used not only for fast roughness measurements, but also for the measurement of mechanical parameters.

The metrological properties of these sensors were investigated showing a noise level of the sensors of only 50 pm/$\sqrt{Hz}$ if the sensor is not in contact with a surface. This value increases on measurement machines due to additional noise sources to a value less than 3 nm in a bandwidth of 10 kHz. It also increases further by a factor of 3 when increasing the measurement speed from 20 µm/s to 1 mm/s. Measurements on an optical flat revealed arithmetical mean roughness values of *Ra* = 4 nm and maximum height values of *Rz* = 27 nm. Increasing the static probing force from 53 µN to 1053 µN during measurement also led to an increase in measured roughness by a factor of 3.

One reason for the increase in measured sensor noise when increasing measurement speed and probing force is the low damping ratio D = 1.2∙10^−3^ of the microprobes, which needs to be increased to obtain critical damping.

For very accurate form measurements with these microprobes, the tip shape has to be known. A new silicon tip-testing standard with rectangular grooves of different width from 0.3 µm up to 3 µm was tested for this purpose, but also to measure tip wear. Two methods to determine the tip shape can be used. The first method measures the sinking-in of the microprobe tip into the grooves and the second method consists of a direct imaging of the tip shape at the edges of the rectangular grooves. Future comparison measurements with other traceable methods are necessary to determine the measurement uncertainty of these new methods.

Tip wear of the integrated silicon tips turned out to be considerable for high scanning speeds of 15 mm/s on conventional roughness standards. This wear could easily be measured with the new tip-testing standard. The maximum height roughness parameter *Rz* turned out to be the most suitable for the detection of wear.

To reduce tip wear diamond tips with 2-µm radius and 90° cone angle were glued to the microprobes and have been tested successfully.

The static probing force must be increased in order to preserve the probes from tip flight at high traverse speeds. Different methods currently available for measuring the probing force were briefly summarized. With these methods, uncertainties of the probing force of 1% can be achieved.

Furthermore, the article presented the currently available devices on which microprobes can be used. On the one hand, the PTB Profilescanner allows roughness measurements of up to 1 mm/s that can be carried out in a measuring range of 0.8 mm × 0.8 mm × 0.25 mm. In addition, Breitmeier´s Micro Profiler probe head with integrated feed unit for measuring speeds up to 5 mm/s, is very compact and can be integrated into manufacturing machines.

Experimental results on fast roughness and shape measurements up to 1 mm/s traverse speed inside micro-nozzles like critical flow Venturi nozzles and diesel injection nozzles were presented.

The first experimental results were obtained using the contact resonance method to measure the indentation modulus of polymer surfaces. Further research is necessary to understand and model this method, aiming for small measurement uncertainties.

## Figures and Tables

**Table 1:** Measured roughness parameters for a tilted optical flat (100 µm) and for one that is not tilted for four different traverse speeds (standard deviation in parenthesis, λc = 80 µm and λs = 2.5 µm).

Traverse	No Tilt/100 µm Tilt	
Speed (µm/s)	*Rz* (nm)	*Ra* (nm)
20	27(6)/131(30)	3.7(0.6)/19(4)
100	54(11)/201(63)	5.6(0.9)/24(8)
500	71(21)/196(68)	11(2)/23(3)
1000	63(21)/160(30)	9.4(1.6)/23(2)

**Table 2:** Measured roughness parameters for four different traverse speeds on a well-aligned roughness standard (RN6531) that is not tilted and on the same standard inclined by 100 µm (λc = 80 µm and λs = 2.5 µm).

Traverse	No Tilt/100 µm Tilt	
Speed (µm/s)	*Rz* (nm)	*Ra* (nm)
20	654(156)/854(113)	111(26)/130(13)
100	649(153)/857(103)	109(27)/130(14)
500	617(160)/893(129)	108(28)/132(16)
1000	605(152)/833(156)	107(23)/129(22)

**Table 3:** Specifications of PTB’s Profilescanner for the traceable measurement of high aspect microstructures.

Measurement range (*x* × *y* × *z*)	800 µm × 800 µm × 250 µm
Stage travelling distance (*x* × *y* × *z*)	50 mm × 50 mm × 12 mm
Rotary stage continuous	360°
Maximum artefact size (*x* × *y* × *z*)	80 mm × 100 mm × 100 mm
Minimum probing force	1 µN
Maximum scanning rate	1 mm s^−1^
Maximum sampling rate	19.2 kHz
Data points per scan line	65 536 maximum
Available microprobe length	1.25, 3 and 5 mm
Tip radius Si/diamond	< 0.1 µm/2 µm

**Table 4:** Specifications of the 1.25-mm in length piezo-resistive silicon microprobes.

Parameter	Value
Cantilever length, width, height	1.25 mm/30 µm/25 µm
Operation temperature	−20 °C … +80 °C
Sensitivity	(4.0 ± 0.3) nm/µV/V
Temperature coefficient	(0.31 ± 0.1) %/K
	(−2 ± 10) µV/V/K
Noise (1.6 kHz bandwidth, amplifier 0.4 µV/V)	2.4 nm
Light susceptibility (cold light 0.1 mW/cm^2^)	9.2 nm
Tip height/radius	(15 – 25) µm/< 100 nm
Cone angle	(40 ± 2) °
Tip wear (100 µN static force)	0.1 nm/m
Fracture limit axial/lateral/vertical	170/520/470 µm deflection
Resonance frequency	46.4 kHz
